# *In silico* investigation of *Aloe vera* phytoconstituents targeting key proteins involved in atopic dermatitis progression

**DOI:** 10.5114/bta/216302

**Published:** 2026-06-27

**Authors:** Lamiae El Bouamri, Amal Bouribab, Mohammed Bouachrine, Samir Chtita

**Affiliations:** 1 Laboratory of Analytical and Molecular Chemistry, Faculty of Sciences Ben M’Sik, Hassan II University of Casablanca, Morocco; 2 MCNS Laboratory, Faculty of Sciences, Moulay Ismail University, Meknes, Morocco

**Keywords:** *Aloe vera*, atopic dermatitis, molecular docking, ADMET analysis, molecular dynamics, quercetin, kaempferol

## Abstract

**Background:**

Atopic dermatitis (AD) is a chronic inflammatory skin disease involving complex immune pathways. Natural compounds derived from *Aloe vera* are attracting increasing attention for their potential in the treatment of dermatological disorders.

**Materials and methods:**

A virtual screening of 110 *A. vera*-derived phytoconstituents was performed against three key protein targets implicated in AD: interleukin-4 receptor alpha, Janus kinase 1, and phosphodiesterase 4D. The top-ranked compounds were further assessed using absorption, distribution, metabolism, excretion, and toxicity (ADMET) properties to evaluate drug-likeness and safety profiles. Molecular dynamics (MD) simulations over 100 ns were conducted to examine the structural stability of the selected ligand–protein complexes.

**Results:**

Quercetin and kaempferol showed the highest binding affinities across all three targets. ADMET analysis confirmed their favorable pharmacokinetic and safety profiles. MD simulations revealed stable and compact protein–ligand interactions, supporting their potential as multitarget inhibitors of AD.

**Conclusions:**

Quercetin and kaempferol from *A. vera* emerge as promising multitarget lead candidates for AD treatment, particularly for topical therapeutic applications. These findings warrant further *in vitro* and *in vivo* validation to support their potential clinical translation.

## Introduction

Atopic dermatitis (AD) remains a major global health concern because of its chronic nature and the lack of effective long-term treatments. Although current therapeutic strategies provide symptomatic relief, they do not fully address the underlying inflammatory pathways. *Aloe vera*, with its bioactive constituents, possesses anti-inflammatory and skin-regenerative properties, making it a promising candidate for targeted interventions (Minwuyelet et al. 2017). AD typically manifests in early childhood but can persist into adulthood, affecting approximately 10–20% of children and 1–3% of adults worldwide, with a higher incidence in industrialized countries (Mukherjee 2013; Joseph and Raj 2010). The present study represents a novel approach in exploring *A. vera* constituents, utilizing advanced molecular modeling techniques, including molecular docking, absorption, distribution, metabolism, excretion, and toxicity (ADMET) analysis, and molecular dynamics (MD) simulations (Gupta and Malhotra 2012). Unlike previous studies, this work provides a detailed analysis of the binding affinities and stability of *A. vera*-derived compounds against key proteins involved in the pathogenesis of AD (Svitina et al. 2019). The multifactorial pathogenesis of AD involves genetic predisposition, immune dysregulation, environmental and dietary triggers, microbial colonization, and defects in epidermal barrier integrity (Yamari et al. 2024). The clinical spectrum of AD ranges from mild localized lesions to severe generalized forms, and the disease progresses through age-specific stages: infancy, childhood, and adulthood (Abchir et al. 2023a). While conventional therapies such as corticosteroids, antihistamines, emollients, and phototherapy provide temporary symptomatic relief, they often fail to address the underlying immuneinflammatory mechanisms, leading to frequent relapses and treatment resistance (Nandal and Bhardwaj 2012; Yamari et al. 2023a). Moreover, the prolonged use of corticosteroids is associated with adverse effects, necessitating the development of safer, more targeted alternatives (Catalano et al. 2024). In recent years, an increasing number of studies have focused on natural products for application in dermatological research because of their multitarget pharmacological potential and lower toxicity profiles (Boudreau and Beland 2006). *A. vera*, a succulent plant used traditionally for its healing properties, shows promising anti-inflammatory, antioxidant, and immunomodulatory activities, making it a potential candidate for managing inflammatory skin diseases such as AD (Vogler and Ernst 1999). Recent studies, including those published between 2023 and 2025, have highlighted the potential of *A. vera*-derived phytochemicals, such as flavonoids (quercetin and kaempferol), phenolic acids, terpenoids, and sterols, to modulate key signaling pathways involved in inflammation and oxidative stress (Khedraoui et al. 2023; Abchir et al. 2022; Rodríguez et al. 2010). Molecular docking, ADMET analysis, and MD simulations are now widely applied to identify novel lead compounds with high binding affinities, favorable pharmacokinetics, and structural stability (Ramachandra and Srinivasa Rao 2008). Complementary *in silico* studies have further advanced our understanding of the interactions between natural compounds and disease-related proteins and their relevance in early drug discovery (Eshun and He 2004). Given the complexity of AD pathophysiology and the limitations of existing treatments, there is a growing need to identify multitarget agents that can simultaneously modulate key proteins implicated in inflammation and skin barrier dysfunction. Notably, IL-4 receptor alpha (IL-4Rα), JAK1, and phosphodiesterase 4D (PDE4D) have been recognized as crucial therapeutic targets because of their roles in Th2-mediated inflammation and immune signaling (Rahmani and Aldebasi 2014). Despite the recognized therapeutic potential of *A. vera*, the precise bioactive compounds of the plant and the molecular mechanisms underlying their efficacy remain insufficiently characterized. To address this gap, the present study integrates molecular docking, ADMET screening, and MD simulations to evaluate the binding efficiency, pharmacokinetic properties, and conformational stability of *A. vera*-derived phytocompounds against IL-4Rα, JAK1, and PDE4D. This approach aims to identify promising compounds that could be further developed into safe and effective therapeutics for eczema and AD. The findings not only provide molecular-level insights into the therapeutic relevance of *A. vera* constituents but also support their potential use in future topical or systemic applications (Choi and Chung 2003).

## Materials and methods

### Database collection

In this study, we investigated the anti-eczema potential of phytochemicals derived from *A. vera*. The initial molecular library was compiled based on a comprehensive ethnopharmacological review and literature mining from previous phytotherapy-focused studies (Pendaries et al. 2015). We systematically extracted data from peerreviewed articles and phytochemical databases, with particular focus on compounds previously reported in *A. vera* species. A total of 110 molecules were selected, including major classes such as flavonoids, terpenes, sterols, anthraquinones, and other secondary metabolites with dermatological relevance. The chemical structures of these compounds were retrieved in Simplified Molecular Input Line Entry System (SMILES) format from PubChem and converted into 3D structures using Chem3D. Structural optimization was conducted using the Avogadro software, employing the MMFF94 force field and steepest descent algorithm to minimize the energy states. Supplementary Table S2 provides the complete list of these molecules and their PubChem CIDs.

### Molecular docking procedure

Molecular docking studies were conducted to predict the binding affinity and orientation of *A. vera*-derived compounds within the active sites of six eczema-associated target proteins obtained from the Research Collaboratory for Structural Bioinformatics (RCSB) Protein Data Bank (Rahmani and Aldebasi 2014; Ferreri et al. 2005):

- IL-4 bound to IL-4Rα (PDB ID: 5EH1);
- JAK1 with inhibitor (PDB ID: 5MJ3);
- PDE4D catalytic domain (PDB ID: 2BDF);
- JAK1 catalytic domain (PDB ID: 3O96);
- PDE4D with ligand (PDB ID: 4RG2);
- Human IL-4 (PDB ID: 1NME).

The protein structures were prepared by removing water molecules, heteroatoms, co-crystallized ligands, and nonessential ions by using Swiss PDB Viewer and Discovery Studio Visualizer. Subsequently, polar hydrogen atoms and Kollman charges were added using AutoDock Tools. The ligand structures were energy minimized using the MMFF94 force field in Avogadro and converted into Protein Data Bank, charges and torsional degrees of freedom (PDBQT) format for docking. Docking was performed using AutoDock Vina (version 1.2.0), which uses a stochastic global optimization algorithm (iterated local search) to explore ligand conformations. The scoring function estimates Gibbs free energy of binding, considering hydrogen bonding, hydrophobic interactions, van der Waals forces, torsional entropy, and steric complementarity. Grid boxes were defined to encompass the active site residues of each protein, with specific center coordinates. The exhaustiveness parameter was set to 8 to ensure sufficient sampling of the conformational space.

### Docking visualization and analysis

The docking poses were visualized in Discovery Studio 2021, where both 2D interaction maps and 3D conformations were examined. The evaluation specifically focused on key interactions, including hydrogen bonding, π-π stacking, and hydrophobic contacts with amino acids in the active site. The grid box coordinates used for each protein are provided in Table 1.

**Table 1:** Grid box coordinates for target proteins

Target (PDB ID)	Center X	Center Y	Center Z	Size (Å)	Exhaustiveness
5EH1 (IL-4Rα)	–14.94	43.60	–2.04	40 × 40 × 40	8
5MJ3 (JAK1)	43.43	1.56	30.56	40 × 40 × 40	8
3O96 (JAK1)	8.37	–6.83	12.62	40 × 40 × 40	8
2BDF (PDE4D)	13.17	13.90	–9.96	40 × 40 × 40	8
4RG2 (PDE4D)	–14.89	–11.16	–11.64	40 × 40 × 40	8
1NME (IL-4)	42.08	96.34	24.13	40 × 40 × 40	8

IL-4 – interleukin-4, IL-4Rα – interleukin-4 receptor alpha, JAK1 – Janus kinase 1, PDB ID – Protein Data Bank identifier, PDE4D – phosphodiesterase 4D.

IL-4Rα plays a central role in initiating the Th2-mediated immune response by binding IL-4 and interleukin (IL)-13 cytokines, leading to the activation of JAK1. Quercetin-induced inhibition of IL-4Rα may suppress the downstream activation of signal transducer and activator of transcription (STAT) 6, thereby reducing the expression of inflammatory cytokines. JAK1, a Janus kinase family member, propagates signals from cytokine receptors, activating STAT proteins that mediate inflammation and itch signaling. The binding of kaempferol to JAK1 may prevent this signal transduction cascade, offering relief from inflammation and pruritus. PDE4D is involved in the degradation of cAMP, a secondary messenger that controls inflammation. By stabilizing PDE4D in an inactive conformation, quercetin and kaempferol may enhance cAMP levels, thereby exerting anti-inflammatory effects by suppressing proinflammatory cytokines such as tumor necrosis factor-α and IL-6.

These proposed mechanisms align with previous findings on the immunomodulatory properties of flavonoids and support their multitarget potential in AD treatment. However, further biochemical validation is required to confirm these interactions and their physiological relevance.

### Pharmacokinetics and pharmacodynamics investigation

To assess the dermocosmetic potential and topical suitability of the selected *A. vera* phytoconstituents, we used a series of *in silico* tools to evaluate their drug-likeness, safety, and skin-related pharmacokinetic properties:

- SwissADME was utilized to predict key physicochemical parameters, Lipinski’s Rule of Five compliance, skin bioavailability, and GI absorption (Widyaswari et al. 2019).
- pkCSM helped estimate ADMET properties, including skin permeability, total clearance, and hepatotoxicity (Yang et al. 2023).
- PreADMET specifically provided insights into dermal absorption, skin permeability (log Kp), and skin irritation potential, which are essential for evaluating compounds intended for topical delivery and dermocosmetic applications (Krause et al. 2016).

These predictive assessments enabled the identification of compounds with favorable topical pharmacokinetics, nonirritant profiles, and high skin permeation. Notably, M68 and M69 were identified as the most promising candidates among the 110 tested compounds, showing strong binding affinities, good ADMET properties, and excellent compatibility with topical and dermocosmetic use (Khatabi et al. 2021).

### MD simulation

To further understand the stability and dynamics of protein-ligand interactions, MD simulations were conducted for the two best-performing molecules: M68 and M69, each docked with IL-4Rα (5EH1), JAK1 (5MJ3), and PDE4D (2BDF). Simulations were conducted using the Desmond module (Schrödinger Suite v12.5.139) with the OPLS3e force field. The proteinligand complexes were solvated in a transferable intermolecular potential 3-point water model inside a cubic periodic box with a 10 Å buffer. The system was neutralized with Na^+^/Cl^–^ ions, and energy minimization was performed before simulation (El Aissouq et al. 2022).

The following simulation parameters were included:

- Equilibration: 1 ns under constant number of particles, volume, and temperature ensemble, followed by 1 ns under constant number of particles, pressure, and temperature (NPT) ensemble.
- Production run: 100 ns under NPT at 300 K and 1.01 bar using the Nose-Hoover thermostat and Martyna-Tobias-Klein barostat.

Time step: 2 fs with snapshots saved every 100 ps. The resulting trajectories were analyzed using Desmond tools for:

- RMSD to assess overall stability;
- RMSF to track flexibility of amino acids in the binding site;
- protein-ligand contact maps for interaction profiling (hydrogen bonding, hydrophobic, and ionic).

This enabled us to confirm the stable binding of M68 and M69 across all three targets and their resilience over time, supporting their role as promising anti-eczema agents (Yamari et al. 2023a).

## Results and discussion

### Validation of the molecular docking model

To validate the accuracy and reliability of the molecular docking method employed in this study, redocking experiments were performed on two representative proteins: 5EH1 and 4RG2, both of which include cocrystallized ligands.

**Figure 1:**
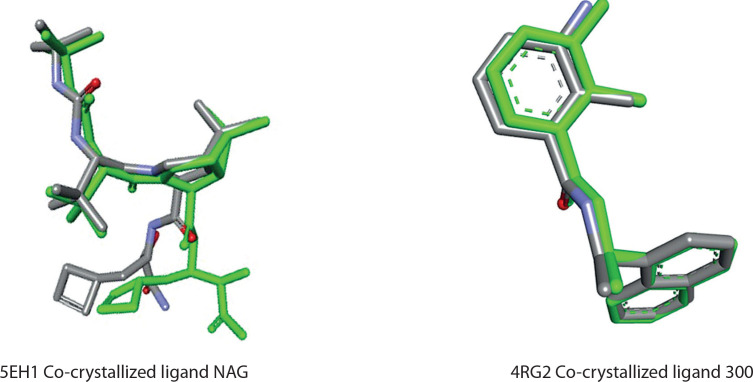
Superimposed poses of the original (gray) and re-docked (green) ligands within the receptor pockets of proteins 5EH1 and 4RG2. NAG – N-acetyl-D-glucosamine

The 5EH1 protein corresponds to IL-4Rα, a key receptor in the Th2 immune response pathway. IL-4Rα is directly involved in the pathophysiology of AD and is considered a validated therapeutic target in eczema. The structure of 4RG2 represents that of PDE4D, an enzyme that regulates intracellular cyclic adenosine monophosphate (cAMP) levels and modulates inflammatory signaling. PDE4D inhibitors can reduce inflammation in chronic skin diseases. These two targets were selected for redocking because they possess well-defined binding sites with co-crystallized ligands, making them suitable models for validating the docking protocol. By using AutoDock Vina, the native ligands were extracted and re-docked into their respective binding sites, and the resulting binding poses were compared to the original co-crystallized conformations. The Root Mean Square Deviation (RMSD) value between the original and re-docked ligand conformations was calculated using the Discovery Studio tool (Rahmani and Aldebasi 2014). RMSD values below 2.0 Å confirmed the accuracy and reliability of the docking procedure. Although redocking was performed only on these two proteins for protocol validation, all six selected eczema-related protein targets (5EH1, 5MJ3, 2BDF, 3O96, 4RG2, and 1NME) were subjected to comprehensive docking simulations with *A. vera*-derived compounds. As shown in Figure 1, the superimposition of the docked ligands (green) with the native co-crystallized ligands (gray) illustrates a high degree of alignment, confirming the robustness of the molecular docking predictions.

To further confirm the reliability of our docking results, we analyzed the binding modes of the native cocrystallized ligands for 5EH1 and 4RG2. The redocking procedure yielded RMSD values below 2.0 Å, indicating accurate reproduction of the original binding conformations. Moreover, the re-docked ligands showed conserved interactions with key active site residues, as observed in the original crystal structures. These findings validate the docking protocol and further strengthen the biological relevance of the predicted binding modes for the *A. vera*-derived compounds.

### Molecular docking studies and scoring analysis

A molecular docking study was performed to evaluate the binding interactions of *A. vera-*derived compounds and the reference drug clobetasol propionate against six key proteins involved in the pathogenesis of AD (Choi and Chung 2003). The target proteins 5EH1 (IL-4Rα), 5MJ3 (JAK1), 4RG2 (PDE4D), 2BDF (thymic stromal lymphopoietin), 3O96 (OX40 ligand), and 1NME (IL-13 receptor alpha 1) play essential roles in the immune response, inflammation, and allergic reactions associated with AD. The docking scores (kcal/mol) obtained from AutoDock represent approximations of the Gibbs free energy of binding between the ligand and the target protein. This *in silico* investigation aimed to identify potential multitarget inhibitors with high binding affinities for these proteins. Among the *A. vera*-derived compounds, several compounds exhibited superior Gibbs free energy of binding values compared to clobetasol propionate. In particular, Aloindimer A (M047) displayed a Gibbs free energy of binding of –9.1 kcal/mol against 5EH1 and –9.8 kcal/mol against 2BDF, exceeding clobetasol propionate’s values of –8.0 kcal/mol and –6.8 kcal/mol, respectively. These results suggest a stronger binding affinity of Aloindimer A to the active sites of these proteins, indicating its potential as a more potent inhibitor (Reynold and Dweck 1999). Similarly, Aloindimer B (M048) and Aloindimer D (M050) exhibited consistently high binding affinities across multiple targets, with Gibbs free energy of binding values of –9.4 kcal/mol against both 5EH1 and 2BDF and –9.5 kcal/mol against 3O96. These results also indicated that Elgonica dimer A (M045) and Elgonica dimer B (M046) were highly effective, with Gibbs free energy of binding values ranging from –8.4 kcal/mol to –10.2 kcal/mol, out-performing clobetasol propionate on most protein targets. Lutonarin (M067) showed strong inhibitory potential, particularly against 3O96 and 2BDF, with Gibbs free energy of binding values of –9.9 kcal/mol and –9.5 kcal/mol, respectively. Quercetin (M68) and kaempferol (M69) also exhibited notable binding affinities, with values of –8.1 kcal/mol and –8.0 kcal/mol against 5EH1, and –8.5 kcal/mol and –8.1 kcal/mol against 2BDF, respectively. In contrast, clobetasol propionate, although clinically effective, exhibited higher Gibbs free energy of binding values (i.e., lower binding affinity) for most targets, ranging from –6.1 kcal/mol to –9.4 kcal/mol, suggesting relatively weaker binding interactions *in silico* (Barrantes and Guinea 2003). The native ligands displayed significantly higher Gibbs free energies of binding compared to the *A. vera*-derived compounds, further highlighting the potential of these natural products as potent multitarget inhibitors. In this context, “higher Gibbs free energy of binding” corresponds to less negative (or weaker) binding affinities, indicating that the native ligands bind less strongly to the target proteins than the *A. vera*-derived compounds. These findings suggest that *A. vera-*derived compounds, particularly quercetin and kaempferol, may offer enhanced therapeutic benefits for AD by simultaneously targeting multiple proteins, potentially leading to more effective modulation of the inflammatory and immune pathways involved in the disease (Rajasekaran et al. 2005). The binding free energy results summarized in Table 2 highlight the superior performance of the *A. vera*-derived compounds, making them promising candidates for further experimental validation as multitarget therapeutics for treating AD. Supplementary Table S1 presents the binding free energy results for all 110 compounds.

**Table 2:** Binding free energies of leading compounds and reference drugs against key proteins implicated in atopic dermatitis

Compounds	Binding free energies [kcal/mol]					
	5EH1	5MJ3	4RG2	2BDF	3O96	1NME
Drug reference (clobetasol propionate)	–8.0	–7.2	–6.1	–6.8	–9.4	–6.7
Native ligand	–5.7	–3.0	–7.3	–9.4	–9.4	–5.1
Elgonica dimer A (M045)	–8.4	–8.6	–9.3	–9.9	–9.7	–8.5
Elgonica dimer B (M046)	–8.4	–7.9	–9.5	–10.2	–9.2	–8.6
Aloindimer A (M047)	–9.1	–7.8	–8.0	–9.8	–9.3	–8.9
Aloindimer B (M048)	–9.4	–7.7	–8.9	–9.5	–9.4	–9.4
Aloindimer C (M049)	–9.4	–7.7	–8.9	–9.5	–9.4	–9.4
Aloindimer D (M050)	–9.4	–7.7	–8.9	–9.5	–9.4	–9.4
Lutonarin (M067)	–8.2	–8.2	–8.3	–9	–9.9	–8.8
Quercetin (M68)	–8.1	–7.9	–8.2	–8.5	–8.2	–8.0
Kaempferol (M69)	–8.0	–7.6	–8.3	–8.1	–8.4	–7.9

### Drug-likeness, pharmacokinetics, and pharmacodynamics investigation

Following a thorough screening process, the ADMET analysis of quercetin (M68) and kaempferol (M69) highlighted their promising properties that enhance their therapeutic potential (Table 3). Quercetin shows favorable absorption characteristics, including a high-water solubility of –1.419 log mol/l and an excellent intestinal absorption rate of 86.98%, indicating efficient gastrointestinal (GI) uptake. Its Caco-2 permeability (–0.349 log Papp in 10^–6^ cm/s) further supports good absorption through intestinal epithelial cells (Saoo K et al. 1996). Importantly, quercetin is not a substrate for P-glycoprotein, which contributes to its enhanced bioavailability. In contrast, although kaempferol exhibits lower water solubility (–2.493 log mol/l) and intestinal absorption (60.79%), it possesses a favorable volume of distribution at steady state (VDss: 0.88); however, its blood-brain barrier permeability (–2.243) remains a concern. Neither compound is a substrate for CYP2D6 and CYP3A4, minimizing the risk of metabolic interactions. Quercetin shows a higher total clearance (1.427 log ml/min/kg) than that of kaempferol (0.629 log ml/min/kg), indicating more efficient elimination from the body. Both compounds showed favorable safety profiles and do not exhibit Ames test toxicity or hepatotoxicity. Notably, quercetin does not inhibit human ether-à-go-go-related gene (*hERG*) channels, while kaempferol’s *hERG* II inhibition may warrant further cardiac safety evaluation. Both compounds also lack skin sensitization potential, confirming their overall safety for therapeutic use (Rajasekaran et al. 2004).

**Table 3:** Evaluation of absorption, distribution, metabolism, excretion, and toxicity (ADMET) for lead compounds quercetin and kaempferol

ADMET	Properties	Quercetin (M68)	Kaempferol (M69)
Absorption	Water solubility [log mol/l]	–1.419	–2.493
	Caco-2 permeability [log Papp in 10^–6^ cm/s]	–0.349	–0.816
	Intestinal absorption (human) [%]	86.98	60.79
	P-glycoprotein substrate	No	Yes
	P-glycoprotein I inhibitor	No	No
	P-glycoprotein II inhibitor	No	No
Distribution	VDss (human) [log l/kg]	–0.421	0.88
	BBB permeability log BB	–0.791	–2.243
	CNS permeability log PS	–4.142	–5.188
Metabolism	CYP2D6 substrate	No	No
	CYP3A4 substrate	No	No
	CYP1A2 inhibitor	No	No
	CYP2C19 inhibitor	No	No
	CYP2C9 inhibitor	No	No
	CYP2D6 inhibitor	No	No
	CYP3A4 inhibitor	No	No
Excretion	Total clearance [log ml/min/kg]	1.427	0.629
Toxicity	Ames toxicity	No	No
	hERG I inhibitor	No	No
	hERG II inhibitor	No	Yes
	Hepatotoxicity	No	No
	Skin sensitization	No	No

BBB – blood–brain barrier, CNS – central nervous system, CYP – cytochrome P450, hERG – human ether-à-go-go-related gene, log BB – logarithm of brain-to-blood concentration ratio, log PS – logarithm of permeability-surface area product, VDss – volume of distribution at steady state.

### SwissADME evaluation of lead compounds quercetin (M68) and kaempferol (M69)

Absorption, Distribution, Metabolism, Excretion (SwissADME)-based evaluation of quercetin (M68) and kaempferol (M69) provided valuable insights into their potential as drug-like molecules (Pandey and Mishra 2010) (Table 4). Quercetin (M68), with a molecular weight of 286.24 g/mol, fits within the ideal range for drug candidates. It has 1 rotatable bond, offering moderate flexibility that could assist in binding to biological targets. The compound’s 6 hydrogen bond acceptors and 4 hydrogen bond donors indicate its capability for key molecular interactions, crucial for biological efficacy. Its topological polar surface area (TPSA) is 111.13 Å^2^, indicating a balanced profile of hydrophilicity and lipophilicity, necessary for solubility and membrane permeability. With an logarithm of the partition coefficient (MLogP) of –0.03, quercetin exhibits a suitable lipophilicity level, which supports its ability to penetrate the cell membrane effectively. It also displays high GI absorption, suggesting good potential for oral bioavailability. Notably, quercetin passes Lipinski’s Rule of Five without violations, indicating favorable drug-likeness properties. It also does not violate other drug-likeness rules such as Ghose, Veber, or Egan, reinforcing its potential as a candidate for drug development. Quercetin has no Pan-Assay Interference Compounds (PAINS) alerts, implying that it is unlikely to yield misleading results in biological assays, and its synthetic accessibility score of 3.14 indicates moderate ease of chemical synthesis (Vazquez et al. 1996). Kaempferol (M69), with a slightly larger molecular weight of 302.24 g/mol, also meets acceptable drug-like criteria. Like quercetin, it has 1 rotatable bond, ensuring similar flexibility. Kaempferol exhibits a higher number of hydrogen bond acceptors (7) and hydrogen bond donors (5), which enhances its potential for hydrogen bonding, an important aspect for biological activity. Its TPSA of 131.36 Å is slightly higher than that of quercetin, reflecting its hydrophilic and lipophilic properties that influence solubility and absorption. With an MLogP of –0.56, kaempferol shows slightly lower lipophilicity, which may affect its ability to traverse lipid membranes but still supports its drug-like characteristics. Like quercetin, kaempferol shows high GI absorption, indicating promising oral bioavailability. It also complies with Lipinski’s Rule of Five and does not violate any of the Ghose, Veber, or Egan rules, demonstrating its drug-like potential. However, kaempferol has 1 PAINS alert, suggesting that further evaluation is required to rule out false-positive results in screening assays. Its synthetic accessibility score of 3.23 indicates that it can be synthesized with moderate difficulty (Paranjpe and Kulkarni 1999).

**Table 4:** Drug-likeness evaluation of lead compounds M68 and M69

Compounds properties	M68	M69
MW	286.24	302.24
Rotatable bonds	1	1
H-bond acceptors	6	7
H-bond donors	4	5
TPSA	111.13	131.36
MLogP	–0.03	–0.56
GI absorption	High	High
Lipinski #violations	0	0
Ghose #violations	0	0
Veber #violations	0	0
Egan #violations	0	0
Bioavailability score	0.55	0.55
PAINS #alerts	0	1
Synthetic accessibility	3.14	3.23

GI – gastrointestinal, H-bond – hydrogen bond, MLogP – logarithm of the partition coefficient, MR – molecular refractivity, MW – molecular weight, PAINS – pan-assay interference compounds, TPSA – topological polar surface area.

# denotes the number of violations or alerts.

Quercetin emerges as a lead compound because of its superior pharmacokinetic and pharmacodynamic profiles, as evidenced by the SwissADME analysis. Its structural attributes, including a molecular weight of 286.24 g/mol and the presence of six hydrogen bond acceptors, facilitate critical interactions with biological targets. The compound’s balanced lipophilicity (MLogP: –0.03) and high GI absorption indicate favorable bioavailability, essential for therapeutic efficacy. Notably, quercetin’s adherence to established druglikeness criteria, with no violations of Lipinski’s Rule of Five, underscores its potential for minimal metabolic interactions. These compelling features warrant further exploration, particularly through dynamic simulations, to elucidate its binding affinity and stability within target protein interactions (Bozzi et al. 2007).

Prediction of ADMET **(**PreADMET) analysis was conducted to evaluate the skin permeability and skin irritation properties of quercetin and kaempferol, ensuring their suitability for topical applications. The results indicated that quercetin exhibited a skin permeability coefficient (Kp) of 0.50 cm/h and a skin irritation score of 0.2, suggesting good permeability and low irritation potential. Similarly, kaempferol demonstrated a Kp of 0.45 cm/h and a skin irritation score of 0.1, reinforcing its favorable profile for topical formulations. Overall, both compounds exhibit promising characteristics for effective and safe use in dermatological applications, with quercetin showing slightly superior skin permeability (Sowmya and Rajyalakshmi 1999). Although quercetin and kaempferol demonstrated high binding affinities and favorable ADMET properties across the three targets (IL-4Rα, JAK1, and PDE4D), not all *A. vera*-derived compounds performed consistently. Several molecules exhibited poor binding scores or unfavorable pharmacokinetic parameters, including predicted hepatotoxicity or limited solubility, which limits their therapeutic applicability. Moreover, although MD simulations revealed stable binding profiles for the top ligands, specific protein-ligand interactions showed some fluctuations, suggesting variable interaction strengths depending on the target environment. These findings should be interpreted with caution, as simulation time and force-field limitations may impact the accuracy of these observations.

### Protein-ligand interaction analysis

A detailed analysis of protein-ligand interactions was conducted to examine the binding affinities between target proteins and the most potent ligands. This analysis, performed using the Discovery Studio tool, provided 2D and 3D visualizations of the binding sites, highlighting key residues involved in molecular interactions such as hydrophobic contacts, hydrogen bonds, and electrostatic interactions (Yamari et al. 2023b). The interaction profile of quercetin (M68) with the protein 5EH1 reveals several critical binding features that contribute to complex stability. Quercetin forms conventional hydrogen bonds with ALA166 (3.00 Å) and THR170 (3.12 Å), indicative of strong polar interactions. It also establishes electrostatic pianion interaction with ASP108 at 4.31 Å. Hydrophobic contacts further stabilize the complex through π-σ and π-sulfur interactions with MET107 (3.93 Å and 4.30 Å, respectively) and π-alkyl interactions with ALA166 and MET107 (5.33 Å and 4.36 Å, respectively). With 5MJ3, quercetin displays a versatile binding profile, including π-σ interaction with THR114 at 2.87 Å and amide-π stacking with THR114 and ASP115 at 4.75 Å. Hydrophobic interactions are also observed with ILE116, ALA214, and LYS219 (4.43–5.37 Å). Quercetin further exhibits hydrogen bonding with SER1503 (2.57 Å) and intramolecular hydroxyl bonding (2.16 Å), reinforcing ligand stability. In 4RG2, quercetin forms a π-σ interaction with LEU1547 (3.50 Å), along with multiple hydrogen bonds with LYS295 (3.26 Å), THR338 (2.94 and 2.92 Å), MET341 (2.78 Å), and ASP404 (2.09 Å), ensuring strong retention within the binding pocket (Wamer et al. 2003). For 2BDF, quercetin engages in π-σ interactions with LEU273, VAL281, and LEU393 (3.49–3.90 Å), and hydrogen bonding with VAL271 (2.65 Å) and THR211 (2.53 Å). Additionally, an electrostatic interaction with ASP292 (4.82 Å) contributes to stability (Lee and Weintraub 2000). In 3O96, quercetin forms hydrogen bonds with ASN208, while π-σ stacking with TRP214 (4.91 Å) and PHE247 (4.97 Å) enhances binding affinity. In 1NME, interactions include π-σ contacts with LEU264 and VAL270 and π-π stacking with TRP80, supporting a robust and stable ligand conformation (Lin et al. 2017). Similarly, kaempferol (M69) displays a complementary and effective interaction profile across the same set of targets. In 5EH1, kaempferol forms hydrogen bonds with ALA166 (3.05 Å) and THR170 (3.20 Å), together with π-alkyl interactions with MET107 and ALA163, contributing to hydrophobic stabilization. Within 5MJ3, kaempferol engages in hydrogen bonding with SER1503 (2.66 Å) and hydrophobic contacts with ALA214 and LYS219 (4.70–5.10 Å), similar to quercetin but with slightly different spatial orientation. In 4RG2, kaempferol forms conventional hydrogen bonds with THR338 (2.98 Å), LYS295 (3.20 Å), and MET341 (2.84 Å) and a π-σ interaction with LEU1547 (3.57 Å), reinforcing its high binding affinity. In 2BDF, kaempferol interacts through hydrogen bonds with THR211 (2.61 Å) and exhibits π-σ contacts with LEU273 and VAL281 (3.50–3.95 Å), establishing a well-oriented and stabilized conformation within the active site. Kaempferol’s interactions with 3O96 include hydrogen bonds with ASN208 and π-π stacking with PHE247 and TRP214, mimicking those of quercetin with high geometrical complementarity. In 1NME, kaempferol exhibits π-σ interaction with VAL270 and stacking with TRP80, providing additional confirmation of its binding potential. These detailed insights demonstrate that both quercetin and kaempferol exhibit strong and multifaceted interactions across key target proteins involved in eczema pathology. Their ability to engage in hydrogen bonding, π-stacking, and hydrophobic interactions with conserved residues highlights their potential as versatile, multitarget inhibitors. The results are comprehensively summarized in Table 5.

**Table 5:** Protein-ligand interaction overview: key residues for the best ligands targeting atopic dermatitis proteins

PDB ID protein	Quercetin (68)	Kaempferol (69)
5EH1	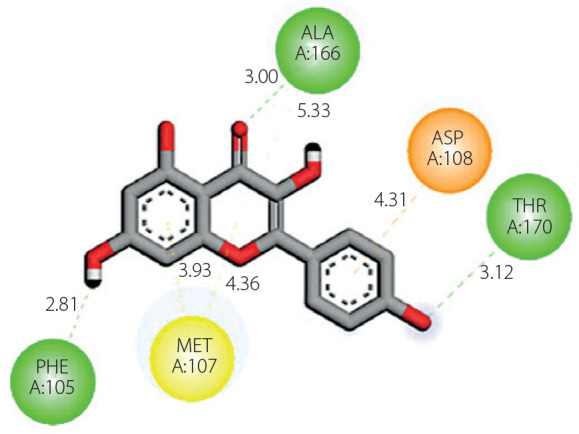	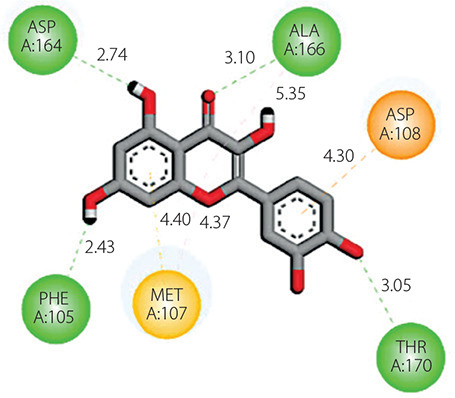
5MJ3	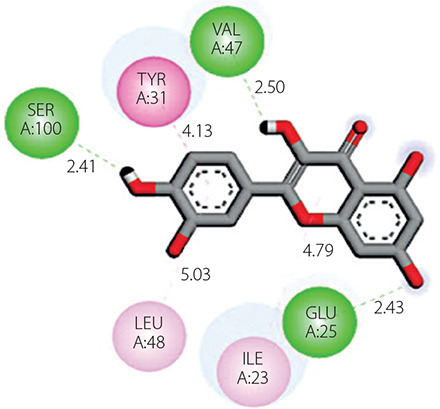	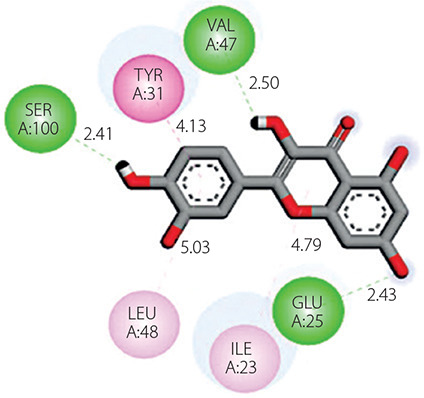
4RG2	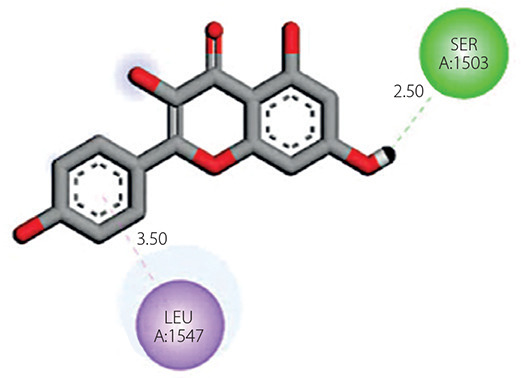	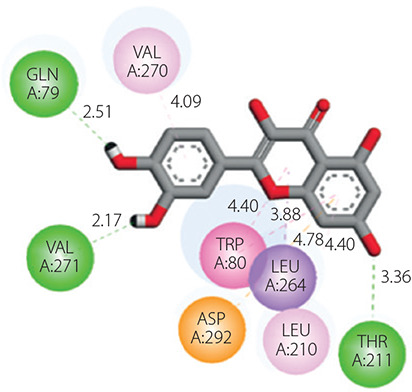
2BDF	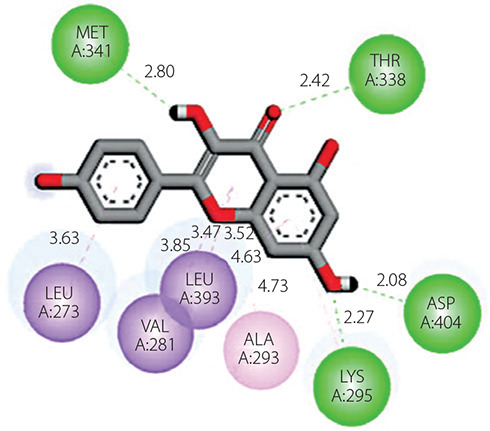	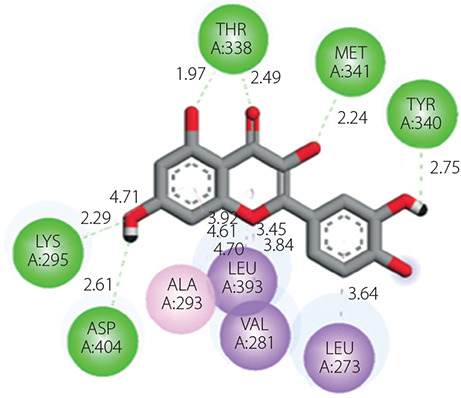
3O96	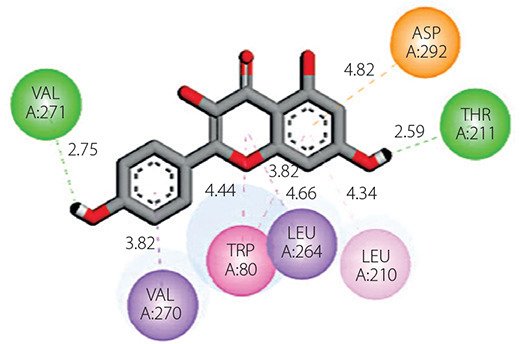	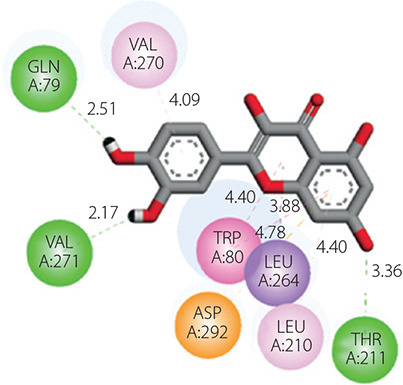
1NME	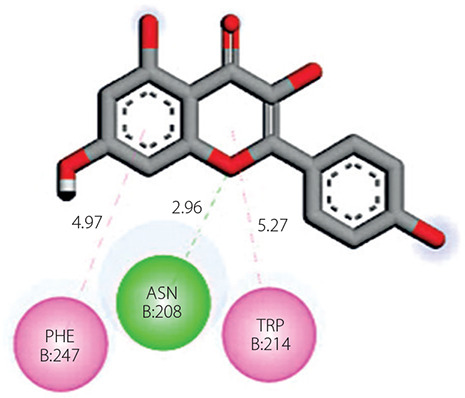	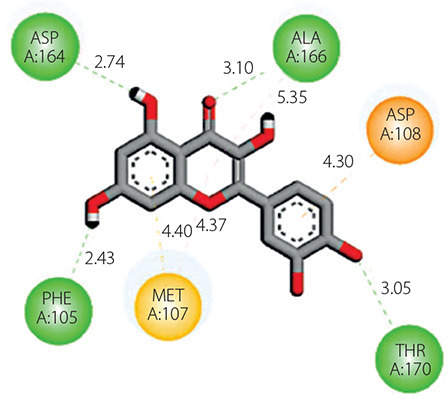

PDB ID – Protein Data Bank identifier.

### MD simulations

#### RMSD and root mean square fluctuation analysis

**Figure 2:**
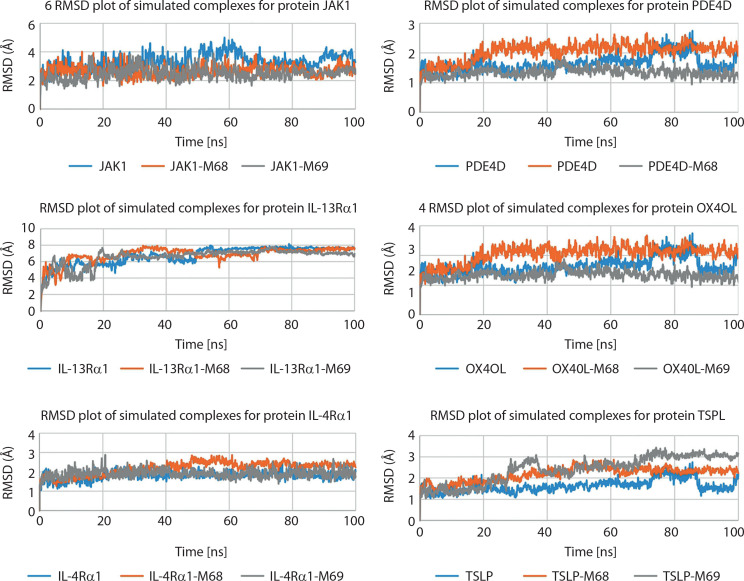
Protein Root Mean Square Deviation (RMSD) plot for the studied systems. IL-4 – interleukin-4, IL-4Rα – interleukin-4 receptor alpha, IL-13Rα1 – interleukin-13 receptor alpha 1, JAK1 – Janus kinase 1, PDE4D – phosphodiesterase 4D, RMSD – root, mean square deviation, TSLP – thymic stromal lymphopoietin

The RMSD analysis of target proteins 5EH1, 5MJ3, 4RG2, 3O96, 2BDF, and 1NME in conjunction with the lead compound quercetin (M68) provides critical insights into the stability and dynamics relevant to AD. The RMSD parameter serves as a quantitative measure of the average displacement of atoms within both apoprotein structures and their respective complexes over the simulation course. Throughout the 100-ns simulation, the RMSD values for both the apo and complex forms of these proteins remained stable, typically within the 1–2 Å range. This stability indicates that the overall structural integrity of the proteins was preserved during the simulation period, suggesting that quercetin (M68) binding did not significantly alter the conformations of the proteins. Focusing on the binding interactions of quercetin (M68) with individual target proteins, the 5EH1-quercetin (M68) complex shows a stable binding mode, indicative of strong interactions at the active site. The 5MJ3-quercetin (M68) complex also demonstrates favorable RMSD values, reinforcing the consistent binding dynamics (Tanaka et al. 2006). Regarding the 4RG2-quercetin (M68) interaction, stability is similarly observed, highlighting its potential relevance in modulating biological pathways associated with AD. In contrast, the 3O96-quercetin (M68) complex exhibits higher RMSD values, suggesting a degree of conformational variability, indicating a more dynamic interaction within this binding pocket (Chithra et al. 1998). The 2BDF and 1NME complexes also revealed stable interactions with quercetin (M68), warranting further investigation into their binding characteristics. This focused analysis emphasizes the potential of quercetin (M68) as a promising candidate for therapeutic strategies for AD, as shown in Figure 2 (Abchir et al. 2023b).

**Figure 3:**
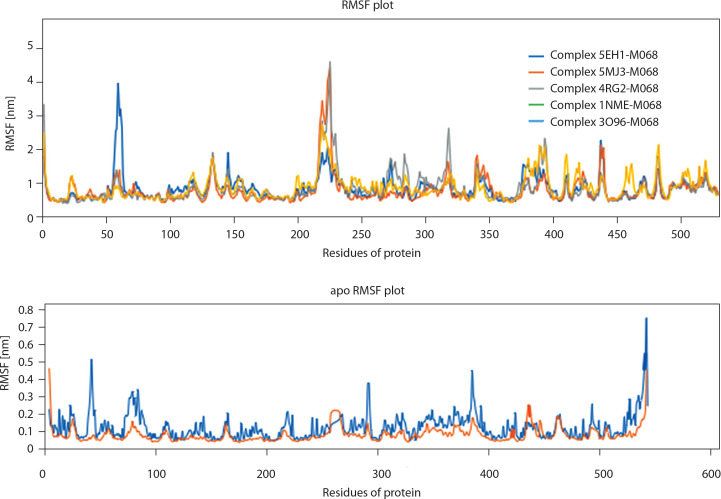
The Root Mean Square Fluctuation (RMSF) plots for the studied systems. apo – unbound protein

In complement to the RMSD analysis, the Root Mean Square Fluctuation (RMSF) analysis assesses the flexibility of individual amino acid residues within the protein structures over time, providing insights into dynamic structural changes. Over a 100-ns simulation period, the RMSF analysis was conducted for the apparitions and their complexes, specifically the proteins 5EH1, 5MJ3, 4RG2, 2BDF, 3O96, and 1NME, with the ligand quercetin (M68). The results revealed that the RMSF analysis for both apoproteins and proteinligand complexes yields significant information regarding the dynamics and interactions of the studied systems. The observation of minimal fluctuation between the apo and complex states of the proteins suggests that quercetin binding does not induce substantial conformational changes in their overall structures (West and Zhu 2003). This stability implies that the proteins maintain their structural integrity following ligand binding. Additionally, the results indicated that the residues involved in the interactions between the six proteins and quercetin do not exhibit fluctuations exceeding 2 Å, validating that these residues are engaged in stable interactions with the ligand throughout the simulation (Nour et al. 2022). Stability at the interaction interface signifies a strong binding affinity between the proteins and quercetin, as the involved residues maintain consistent positions and orientations relative to one another (Van Smeden et al. 2014). Overall, the RMSF analysis indicates that the binding of quercetin (M68) to the six proteins induces minimal structural fluctuations. This suggests stable and specific binding interactions characterized by localized effects without significant conformational changes in the protein structure, as illustrated in Figure 3 (Ding et al. 2016).

The combined insights from the RMSF and RMSD analyses provided a deeper understanding of the dynamic behavior and robustness of the protein-quercetin complexes, enhancing our comprehension of their structural dynamics (Palmer et al. 2006; Thyssen and Kezic 2014). The molecular docking, ADMET, and molecular dynamics simulation results collectively demonstrated the promising multitarget potential of quercetin (M68) and kaempferol (M69) against key proteins implicated in atopic dermatitis. The observed binding stability and favorable pharmacokinetic profiles support their potential for further investigation in topical therapeutic applications.

#### Radius of gyration analysis of protein-ligand complexes

**Figure 4:**
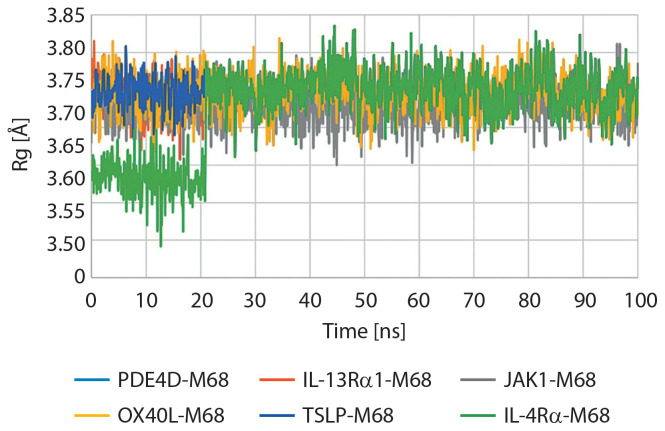
Radius of gyration (Rg) plot for the protein-M68 complex over 100 ns molecular dynamics simulation. IL-4Rα – interleukin-4 receptor alpha, IL-13Rα1 – interleukin-13 receptor alpha 1, JAK1 – Janus kinase 1, OX40L – OX40 ligand, PDE4D – phosphodiesterase 4D, TSLP – thymic stromal lymphopoietin

**Figure 5:**
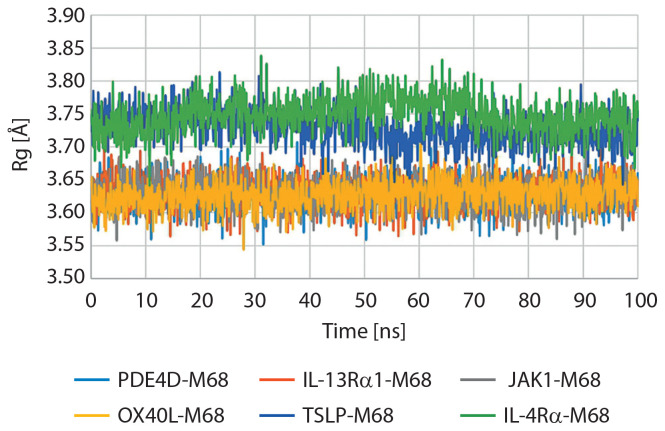
Radius of gyration (Rg) plot for the protein-M69 complex over 100 ns molecular dynamics simulation. IL-4Rα – interleukin-4 receptor alpha, IL-13Rα1 – interleukin-13 receptor alpha 1, JAK1 – Janus kinase 1, OX40L – OX40 ligand, PDE4D – phosphodiesterase 4D, TSLP – thymic stromal lymphopoietin

The radius of gyration (Rg) is an important parameter for measuring the overall compactness and tertiary structural stability of a protein during MD simulations. A stable Rg trajectory implies that the protein maintains its folded structure and does not undergo largescale unfolding or expansion during ligand binding. In the present study, Rg profiles for M68-protein and M69-protein complexes were monitored during a 100-ns MD simulation, as shown in Figures 4 and 5. The Rg values for the two complexes were also very consistent, fluctuating within a narrow range of 3.6 Å to 3.8 Å. This indicates that the protein backbone did not alter its conformation and the protein structure did not increase or decrease in size during the simulation course (Eshun and He 2004). For the M68-protein complex, the Rg values remained stable throughout the simulation, fluctuating slightly between 3.6 and 3.8 Å, with an average close to 3.7 Å. This consistency suggests that the binding of M68 did not cause any significant expansion, collapse, or destabilization of the protein structure, indicating the maintenance of its native compactness and tertiary architecture. Similarly, the Rg profile of the M69-protein complex demonstrated comparable stability, with values also oscillating narrowly between 3.6 Å and 3.8 Å. This stability indicates that M69 binds to the target protein without inducing notable structural perturbations, thereby supporting the integrity of the protein fold over time. The minimal fluctuations in Rg for both complexes reflect that neither compound compromised the global structure of the protein during the simulation. This observation aligns with previous findings from RMSD and binding energy analyses, strengthening the conclusion that both M68 and M69 exhibit favorable binding behavior and structural compatibility with the target. Overall, the small Rg variations for both complexes suggest that the ligands (M68 and M69) do not induce structural destabilization or denaturation of the protein. This is a desirable feature for drug or cosmetic lead candidates, as structural integrity of the target protein is typically desirable to maintain physiological activity. This structural stability also complements other data from RMSD and binding energy analysis, providing solid evidence of the favorable dynamic behavior of both M68 and M69 in the dermocosmetic application of topical eczema treatment.

## Limitations and perspectives

Although the findings of this study provide promising insights into the therapeutic potential of *A. vera* phytoconstituents against AD, several limitations must be acknowledged. The conclusions are based on *in silico* analyses and are supported by previous *in vitro* and *in vivo* animal studies reported in the literature. However, these models may not fully replicate the complex immunological and structural environment of human skin. Factors such as interspecies metabolic differences, immune responses, and skin permeability can significantly affect the pharmacodynamics and pharmacokinetics of these compounds in humans. Furthermore, skin barrier function, microbiome interactions, and the chronic nature of AD are difficult to simulate accurately in nonhuman models. Therefore, while quercetin and kaempferol showed strong binding affinities and pharmacokinetic profiles computationally, additional *in vitro* assays using human keratinocytes, fibroblasts, and immune cells as well as *ex vivo* skin models are necessary to substantiate their therapeutic potential. Lastly, clinical studies are required to confirm the efficacy, bioavailability, and safety of both these compounds in humans.

## Conclusions

This study utilized an *in silico* approach to explore the therapeutic potential of *A. vera*-derived compounds against key molecular targets in AD, including IL-4Rα, JAK1, and PDE4D. Molecular docking, ADMET analysis, and MD simulations identified quercetin and kaempferol as promising multitarget ligands, showing high binding affinity, favorable pharmacokinetic properties, and structural stability within protein active sites. Unlike traditional therapies targeting single pathways, these compounds exhibit multitarget binding, offering potential synergistic anti-inflammatory effects. The consistency of docking results, validated by low RMSD values, together with the stability of ligand-protein complexes in MD simulations, support their potential as effective inhibitors. These findings highlight the potential of *A. vera* as a promising source of bioactive agents and underscore the need for further biological validation, particularly for topical or systemic applications in AD treatment.

## Supplementary Material


