# Supplementary material for: In silico investigation of Aloe vera phytoconstituents targeting key proteins involved in atopic dermatitis progression
Source: BioTechnologia (Pozn). 2026 Jun 27;107(2):121–36. doi: 10.5114/bta/216302 (PMC13409366; doi:10.5114/bta/216302)
Supplement: Supplementary file 1 [file BTA-107-2-216302-s1.pdf]

Table S1: Chemical structures, IUPAC names and 2D structure of aloe vera-derived compounds evaluated as inhibitors in atopic dermatitis therapeutics.

ID	IUPAC names	2D structure
M1	Aloesin	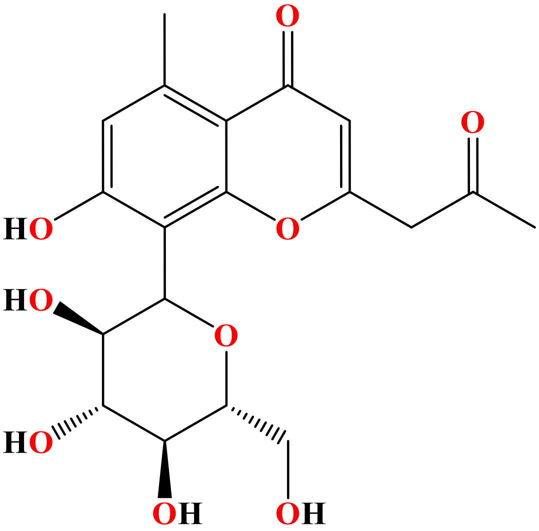
M2	Neoaloesin A	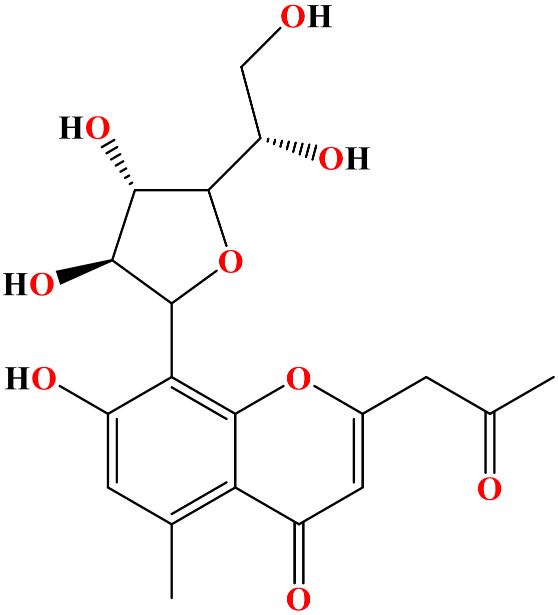
M3	8-C-glucosyl-(R)-aloesol	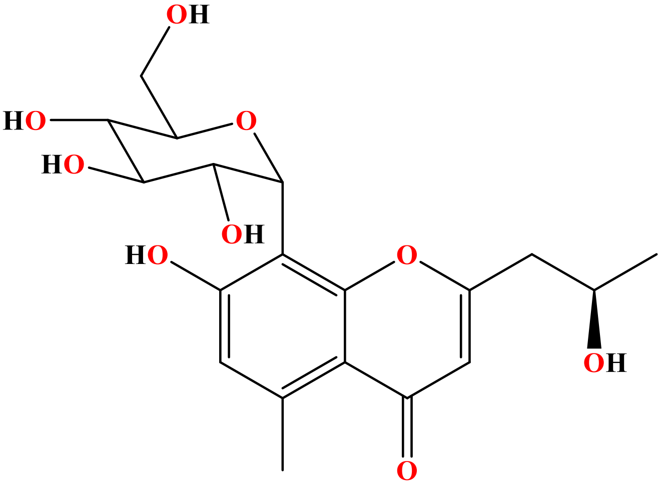

M4	8-C-glucosyl-7-methoxy-(R)-aloesol	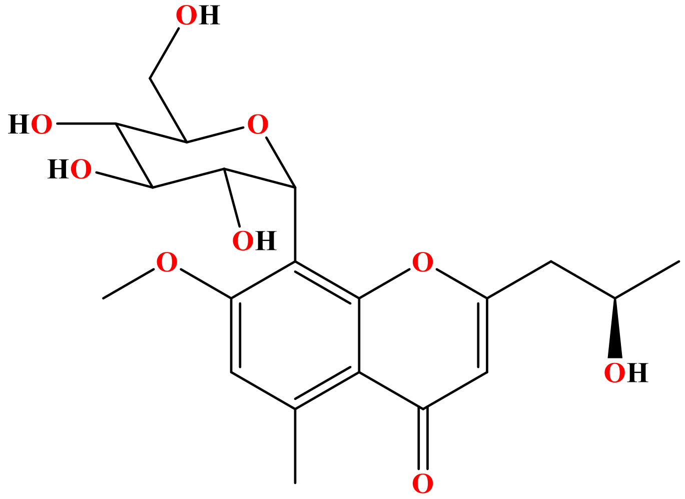
M5	8-C-glucosyl-(S)-aloesol	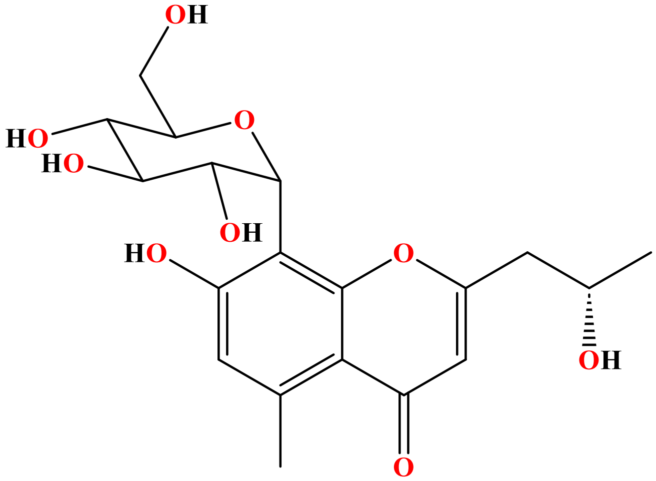
M6	8-C-glucosyl-7-methoxy-(S)-aloesol	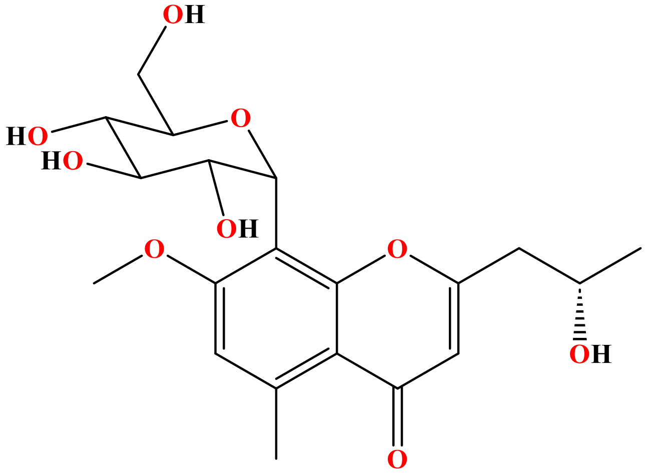

M7	8-C-glucosyl-7-O-methylaloediol	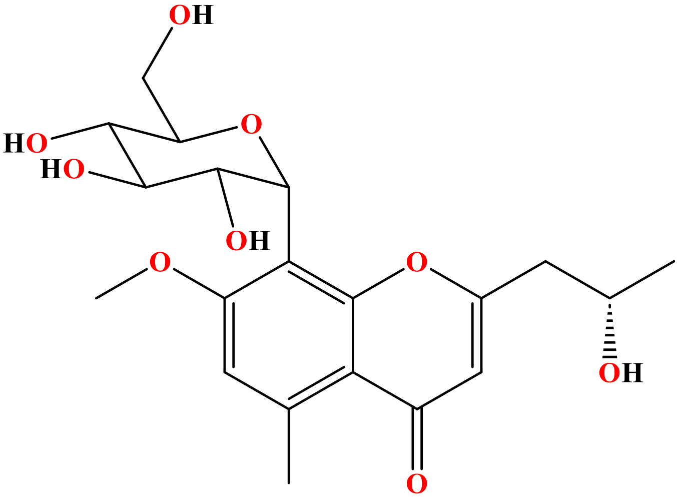
M8	8-glucosyl-(2'-O-cinnamoyl)-7-O-methylaloediol A	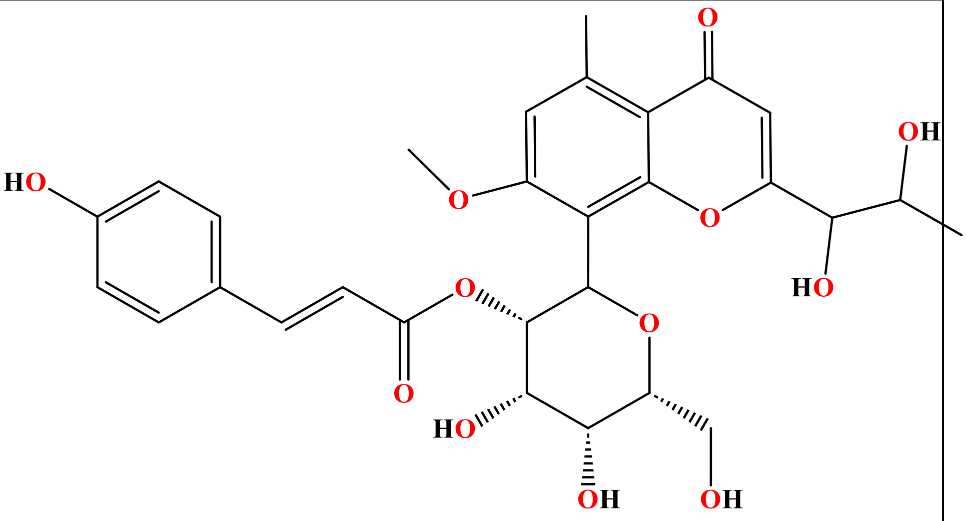
M9	8-glucosyl-(2'-O-cinnamoyl)-7-O-methylaloediol B	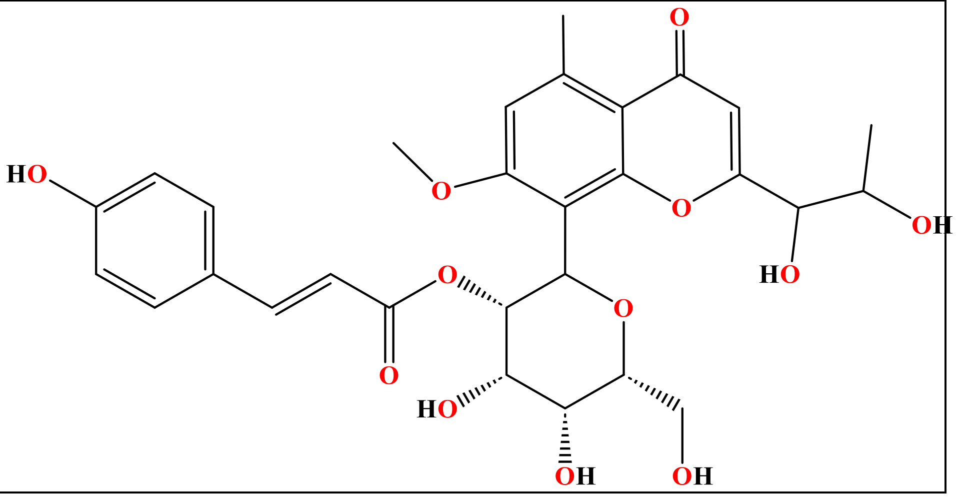

M10	C-2’-decoumaroyl-aloeresin G	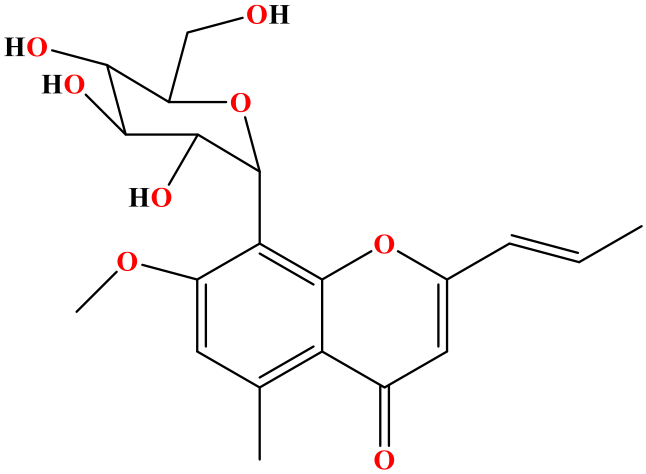
M11	Aloeresin E	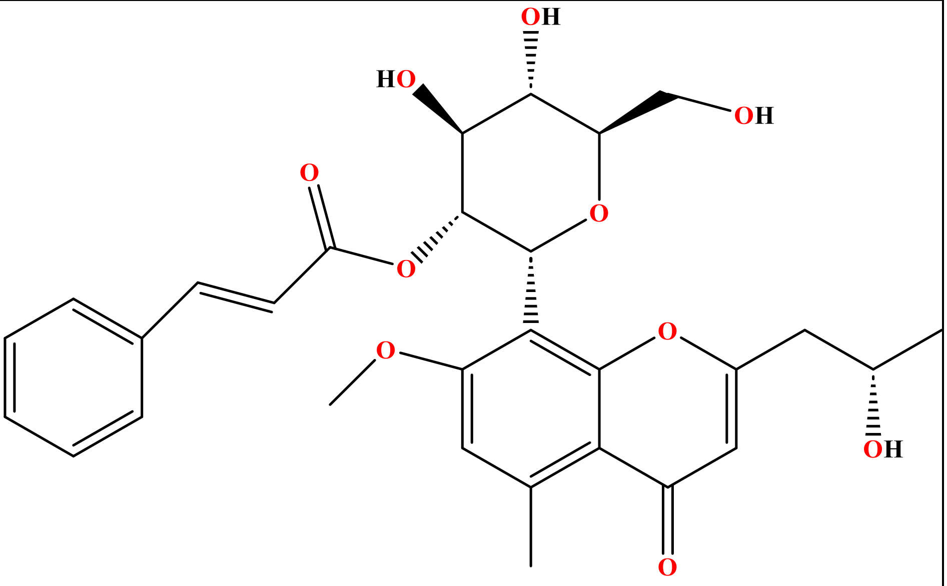
M12	Isoaloeresin D	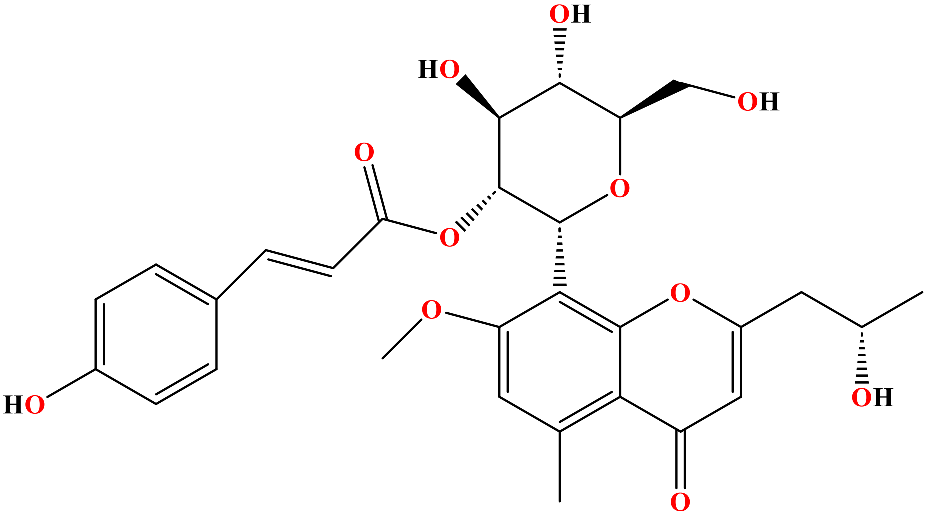

M13	Iso-rabaichromone	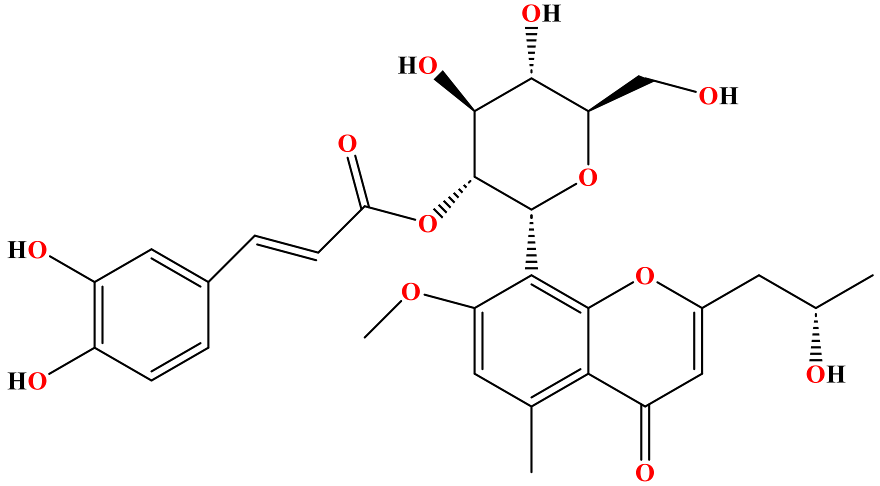
M14	8-[C-β-D-[2-O-(E)-cinnamoyl] glucopyranosyl]-2-[(R)-2-hydroxypropyl]-7-methoxy-5-methylchromone	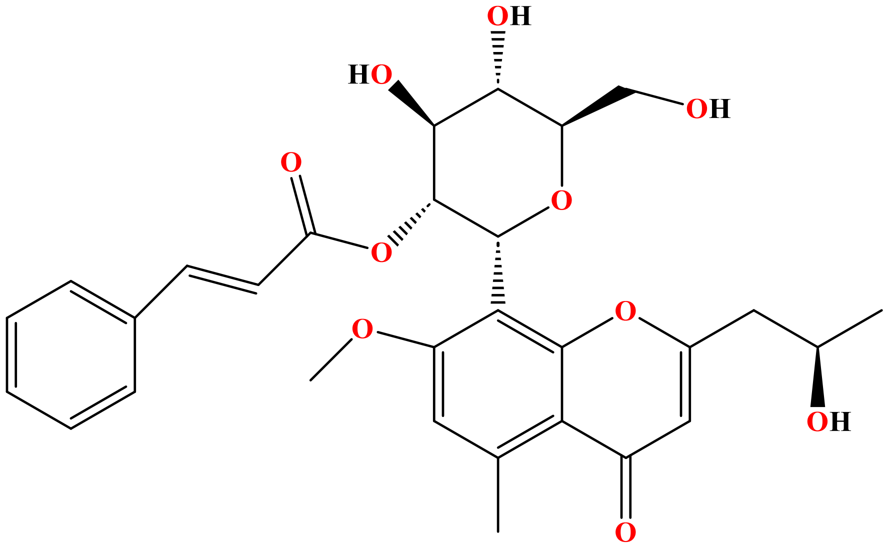
M15	Aloeresin D	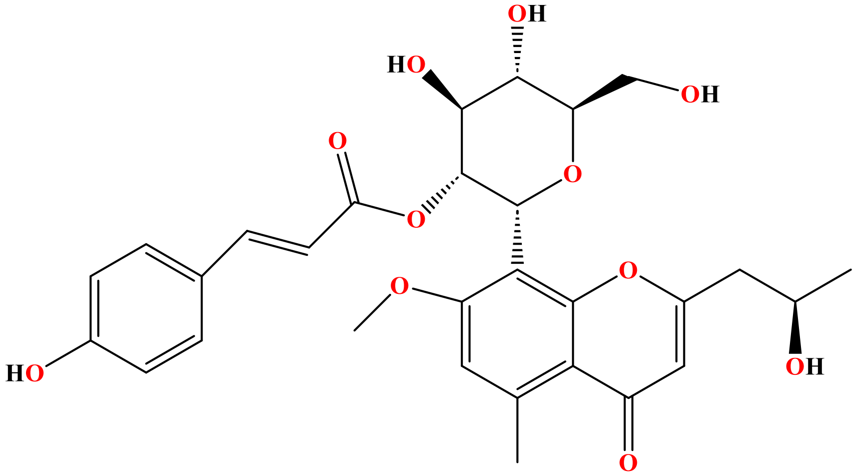

M16	Rabaichromone	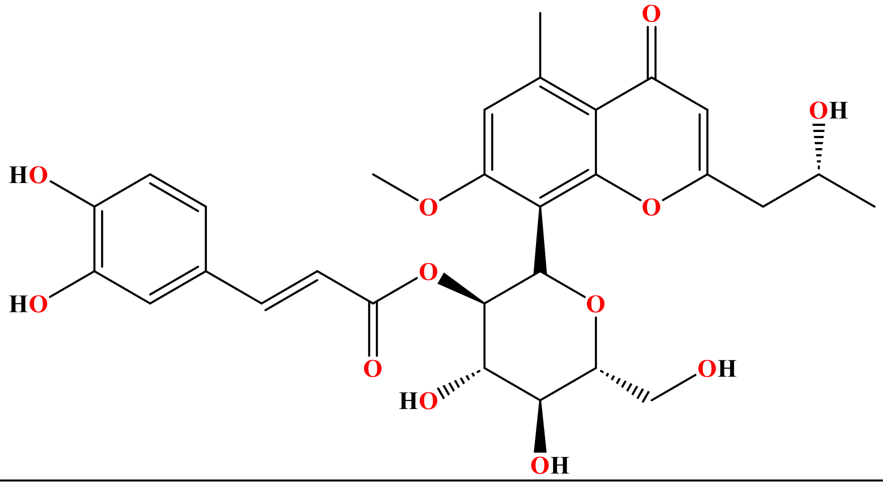
M17	Allo-aloeresin D	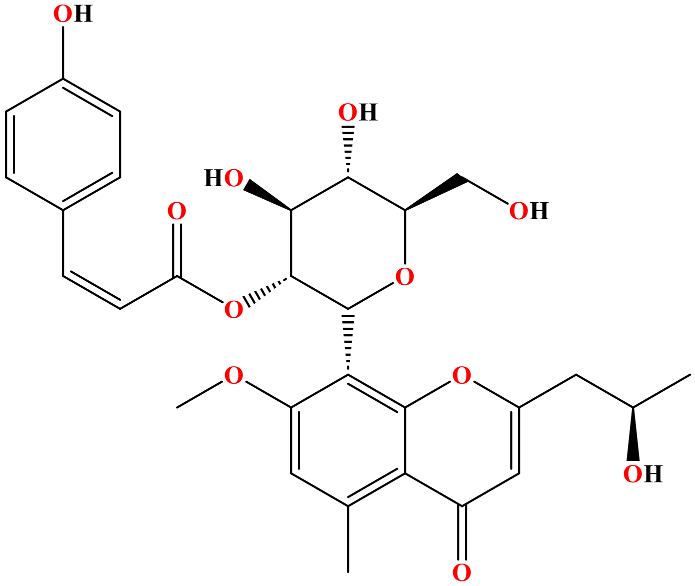
M18	Aloeresin K	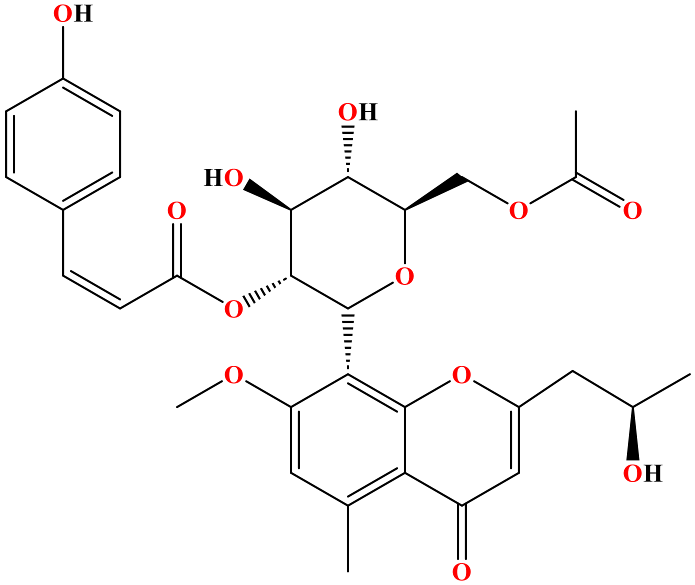

M19	Aloeresin J	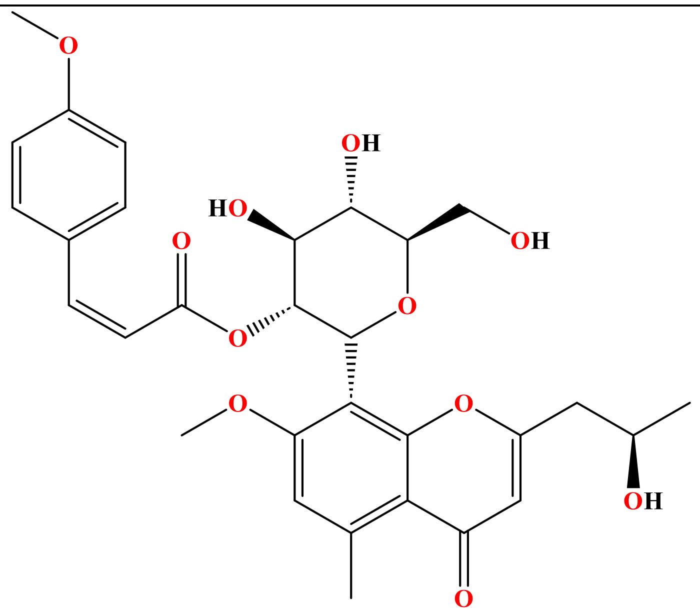
M20	8-C-glucosyl-noreugenin	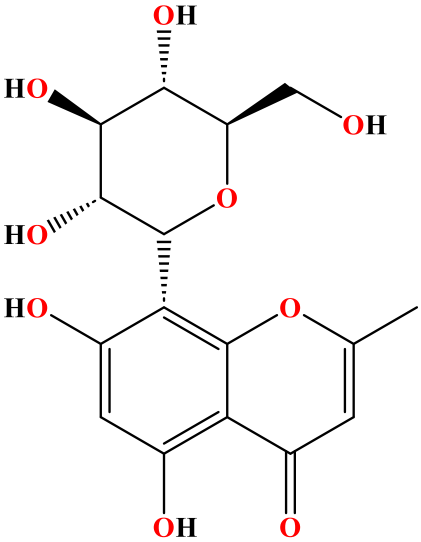

M21	4'-O-glucosyl-isoaloeresin DI	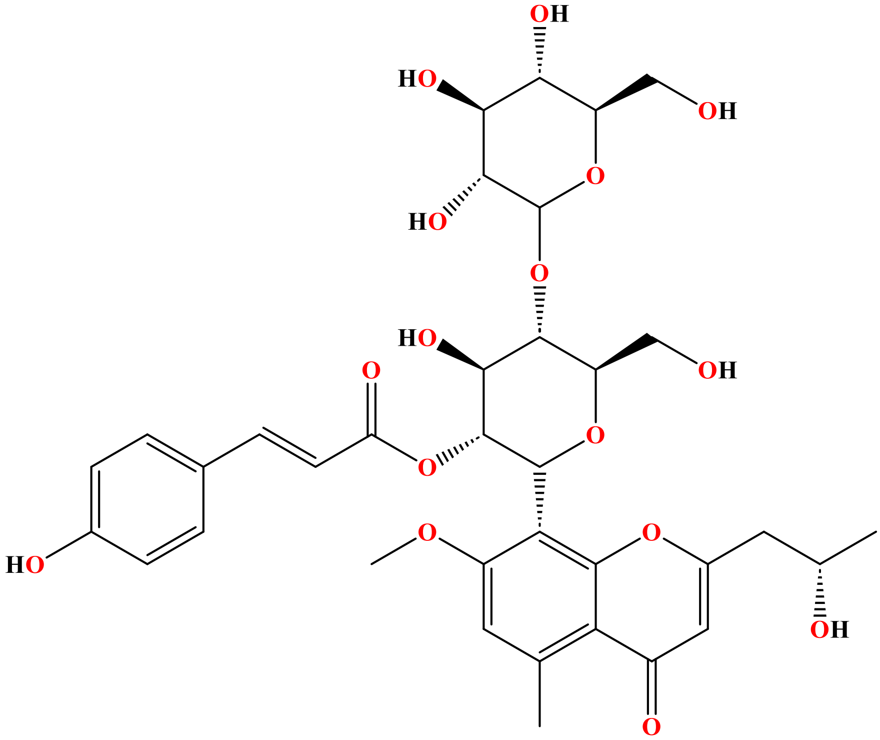
M22	4'-O-glucosyl-isoaloeresin DII	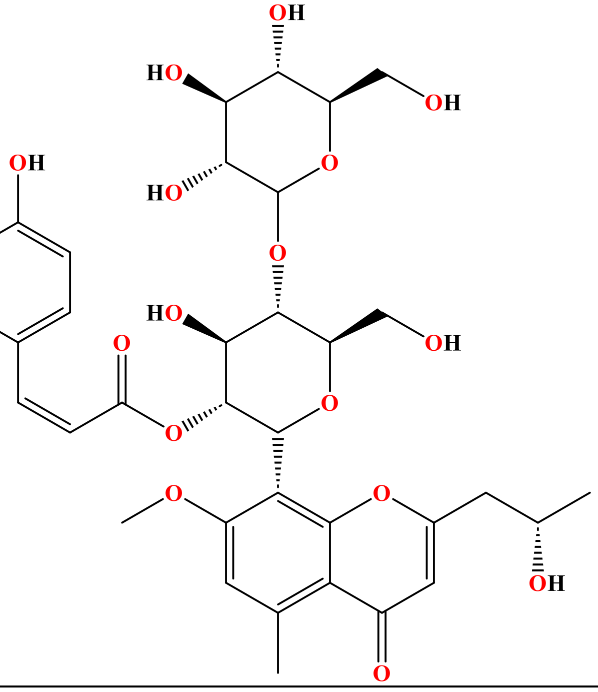

M23	Aloeresin A	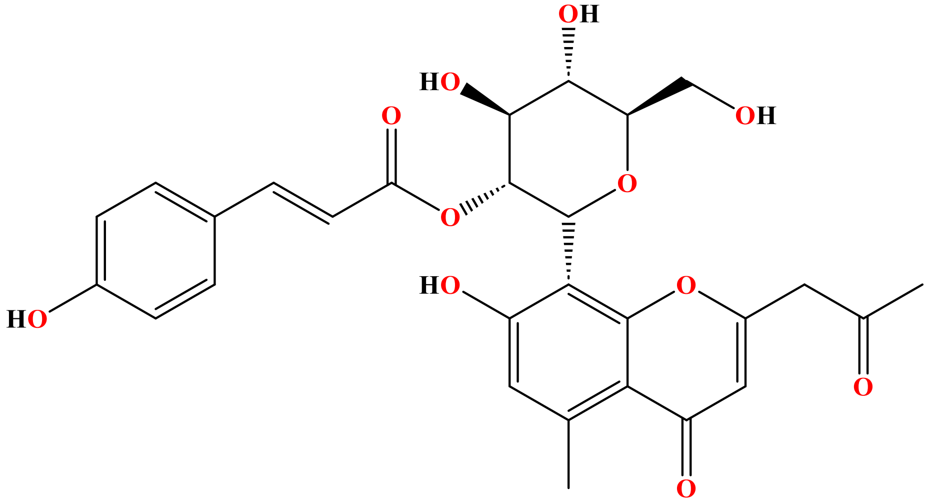
M24	7-O-methyl-aloeresin A	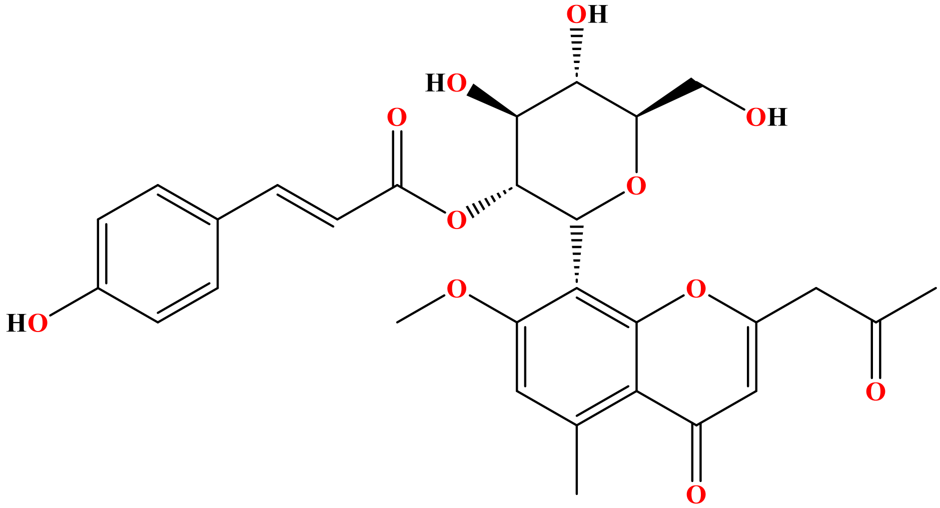
M25	9-dihydroxyl-2'-O-(Z)-cinnamoyl-7-methoxy-aloesin	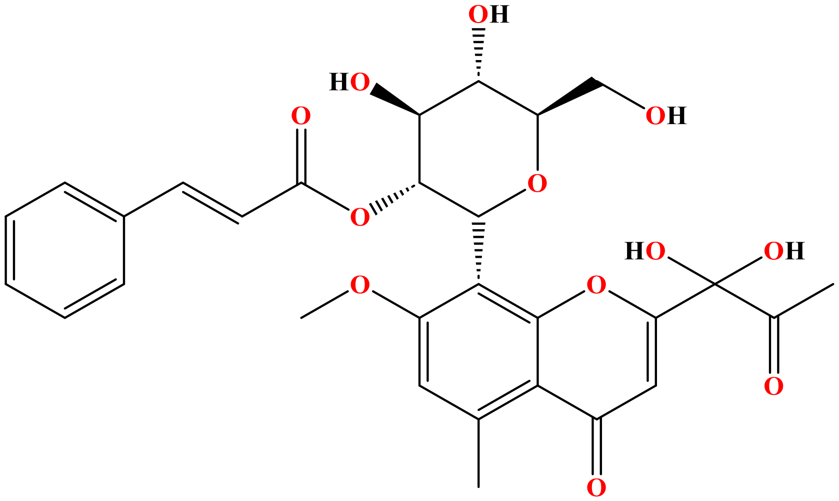

M26	6’-O-coumaroyl-aloesin	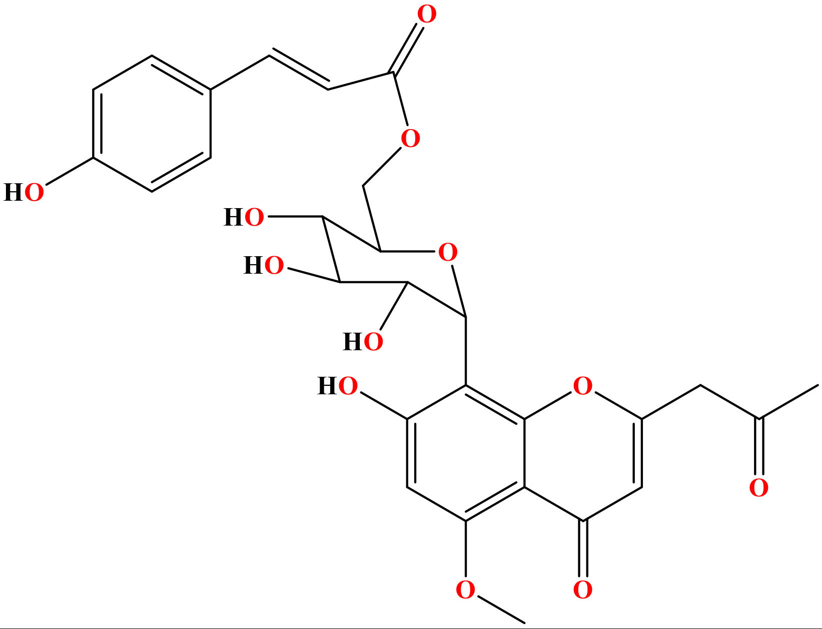
M27	7-methoxy-6’-O-coumaroyl-aloesin	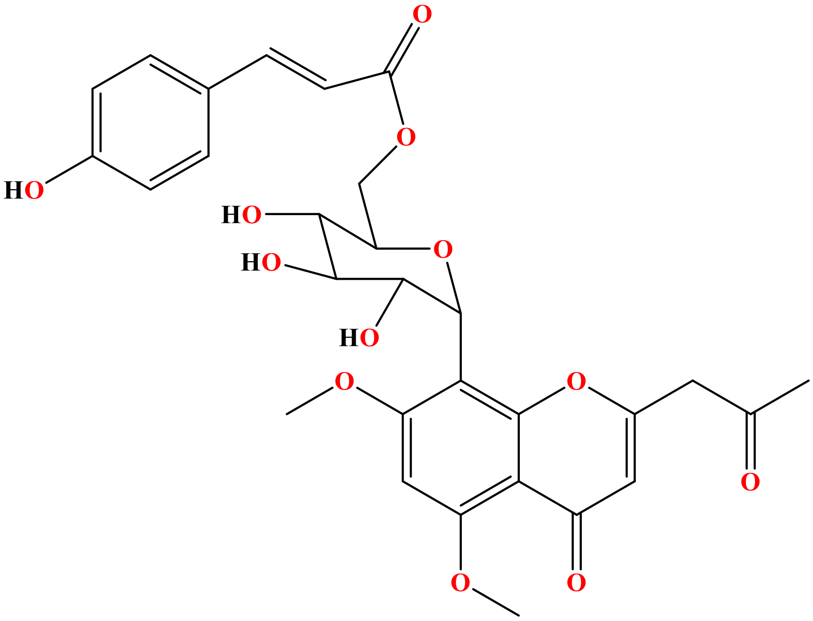
M28	Aloeveraside B	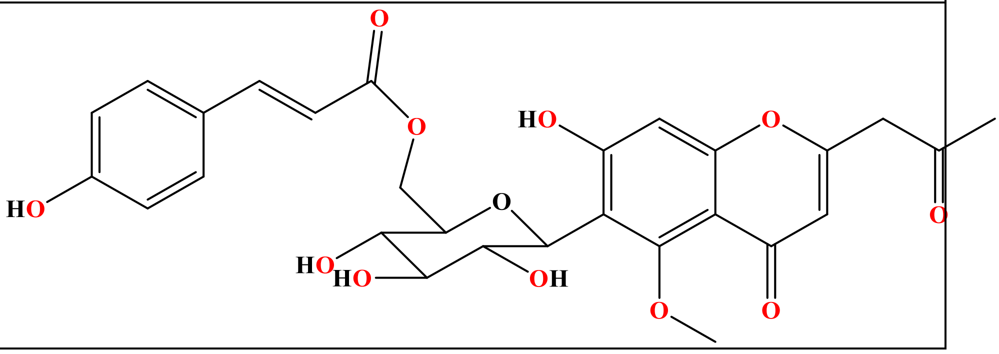

M29	Aloeveraside A	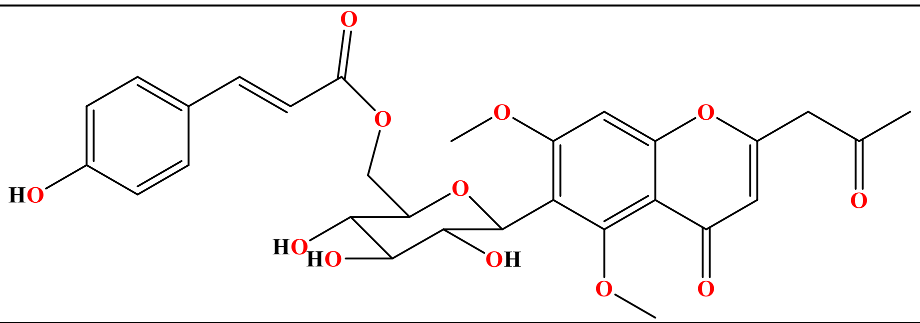
M30	Aloin A	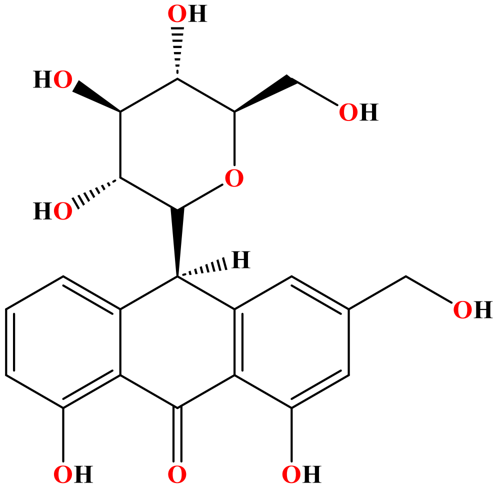
M31	Aloin B	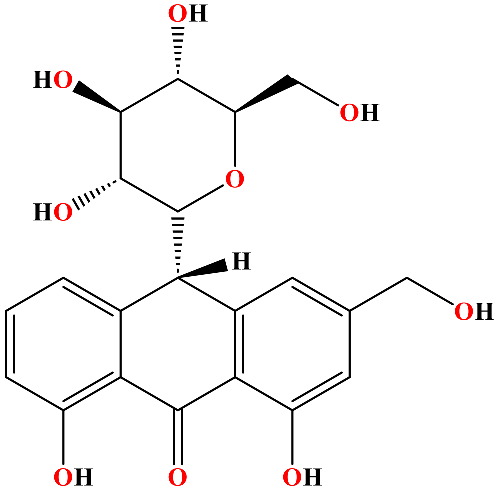
M32	6'-O-acetyl-aloin A	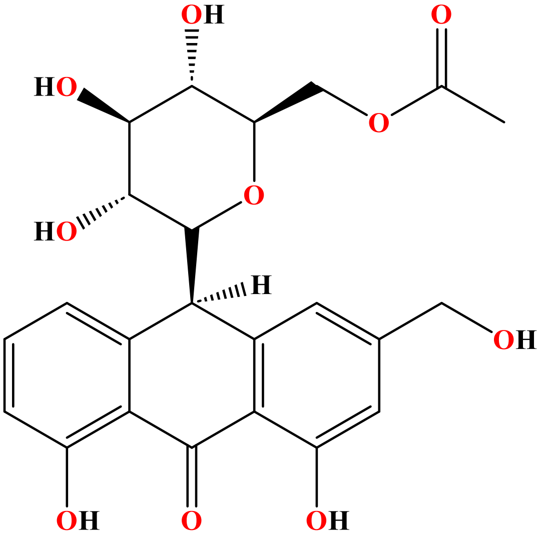

M33	6’-O-acetyl-aloin B	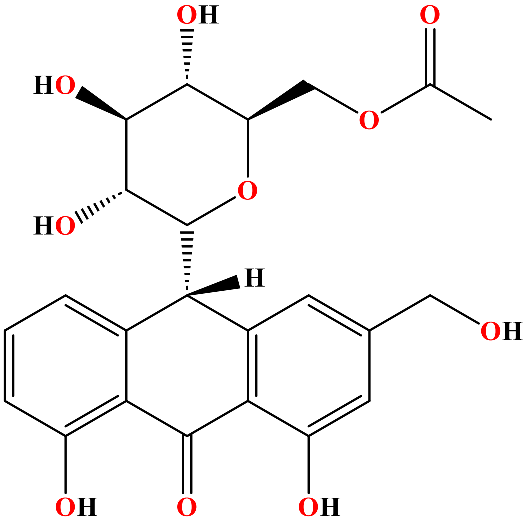
M34	10-hydroxyaloins A	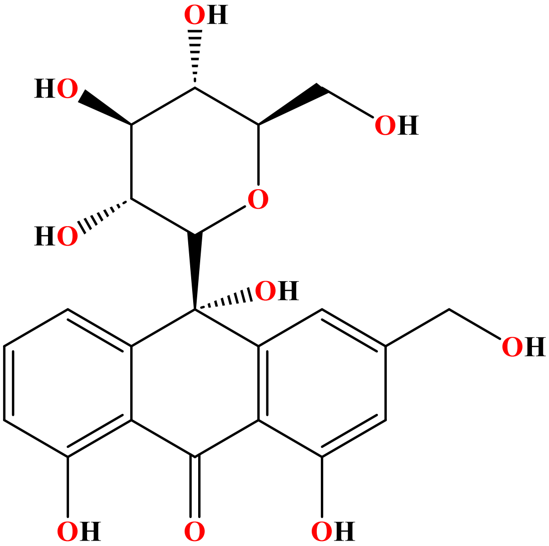
M35	10-hydroxyaloins B	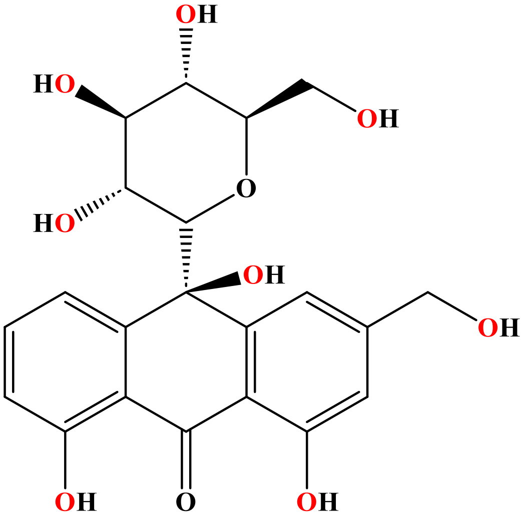

M36	Aloinoside A	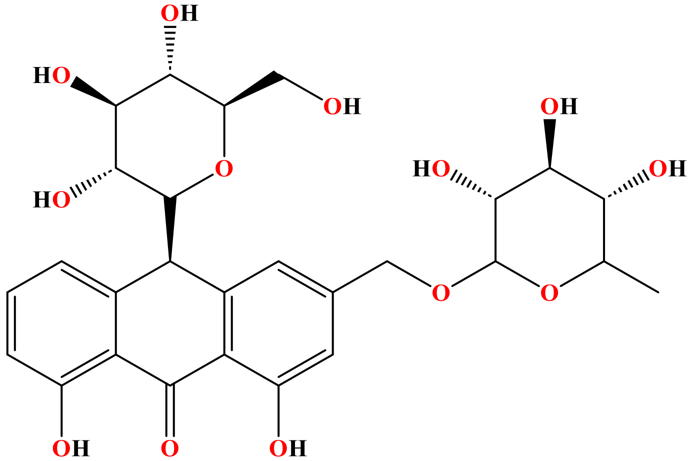
M37	Aloinoside B	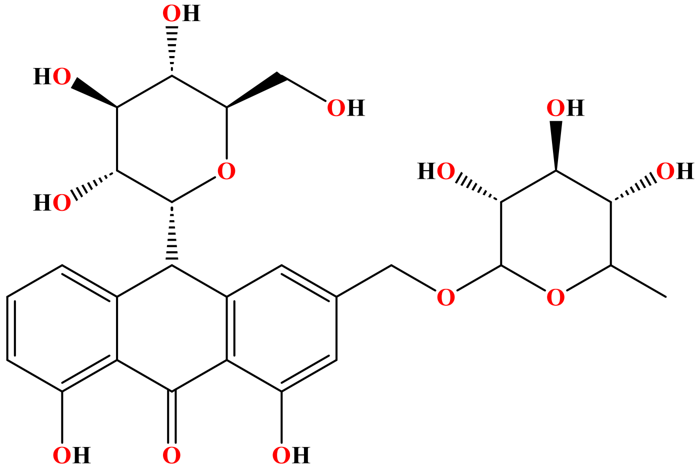
M38	7-hydroxyaloin A	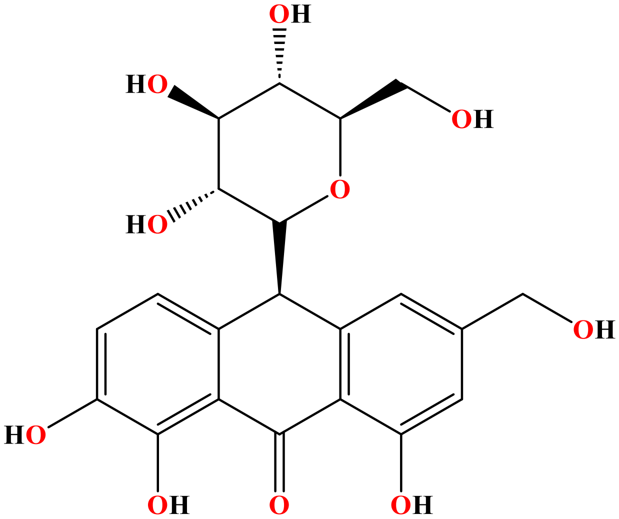

M39	7-hydroxyaloin B	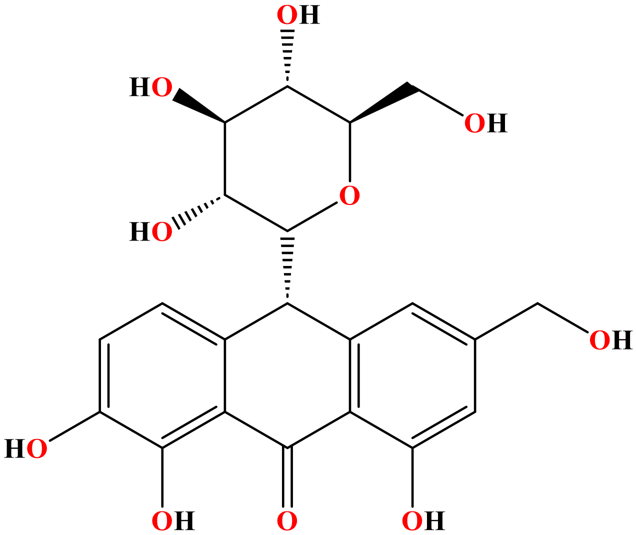
M40	7-hydroxy-8-O-methylaloin A	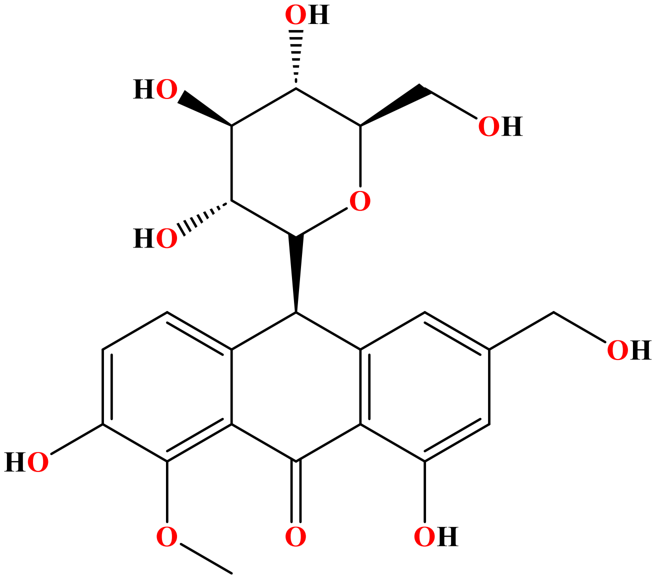
M41	7-hydroxy-8-O-methylaloin B	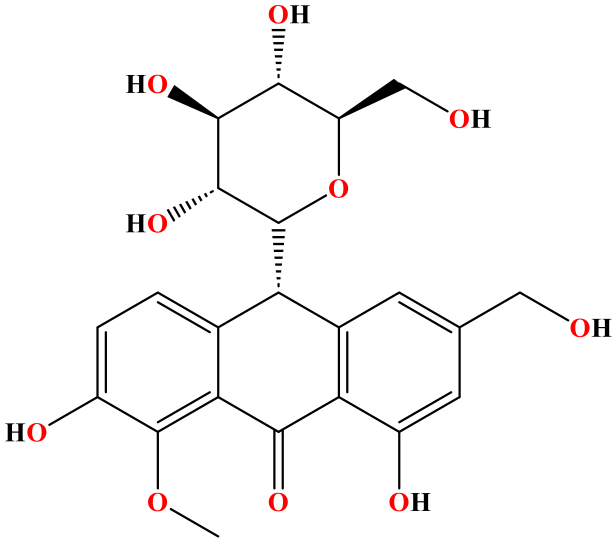

M42	6'-malonylnataloin A	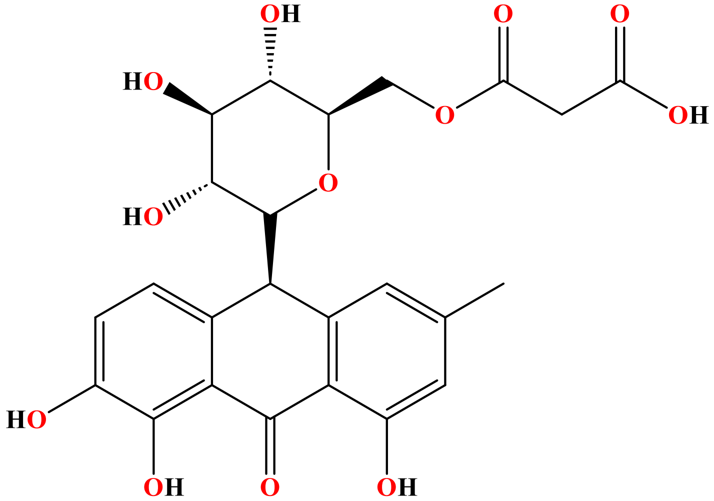
M43	6'-malonylnataloin B	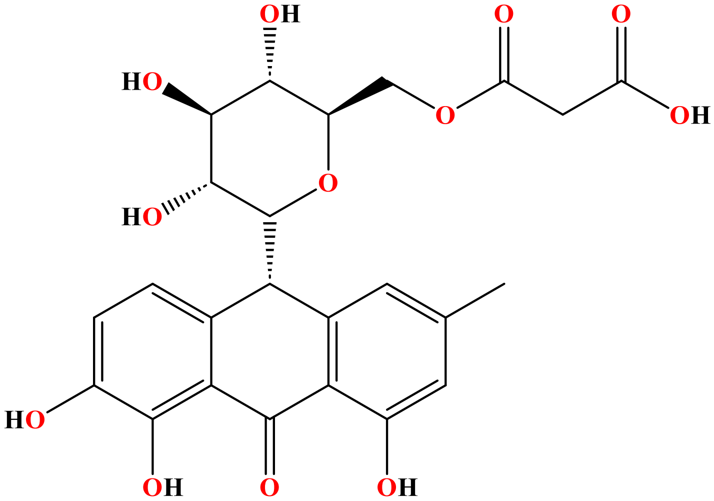
M44	Homonataloside B	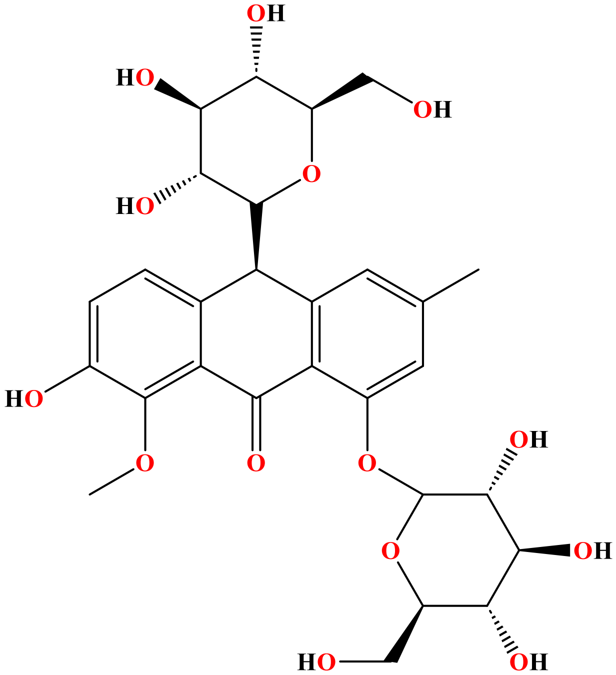

M45	Elgonica dimer A	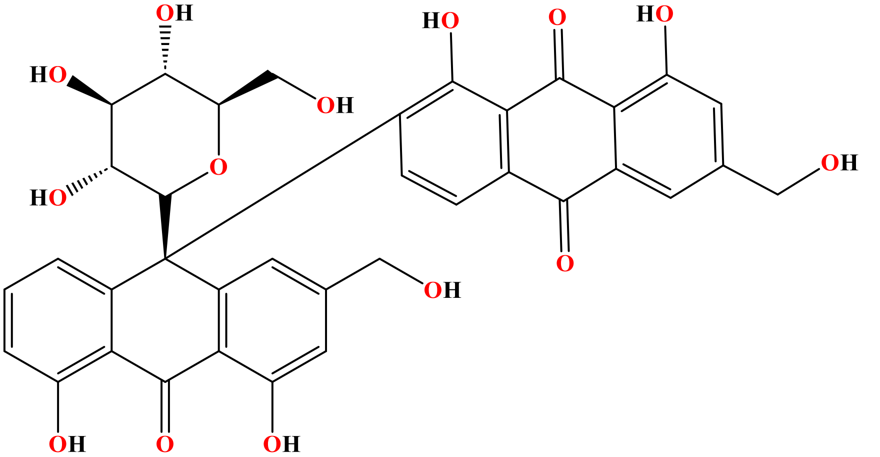
M46	Elgonica dimer B	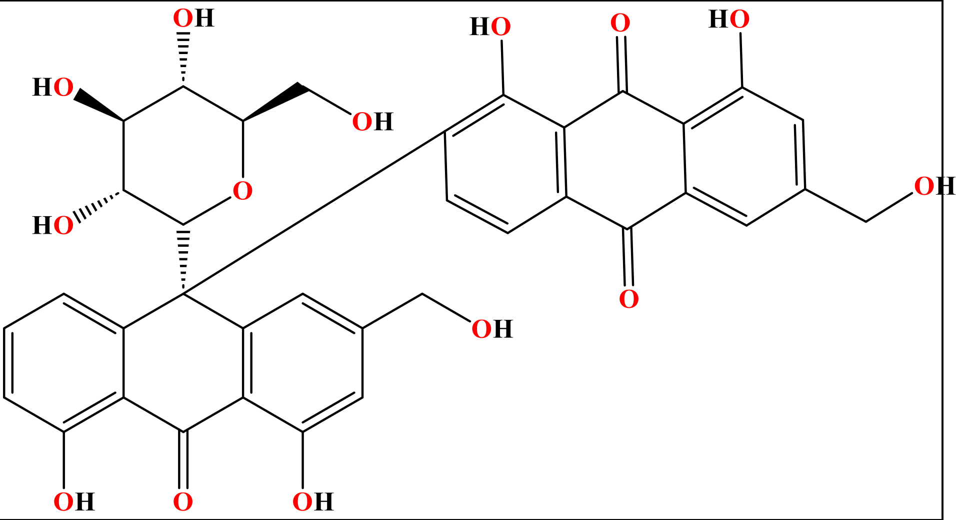
M47	Aloindimer A	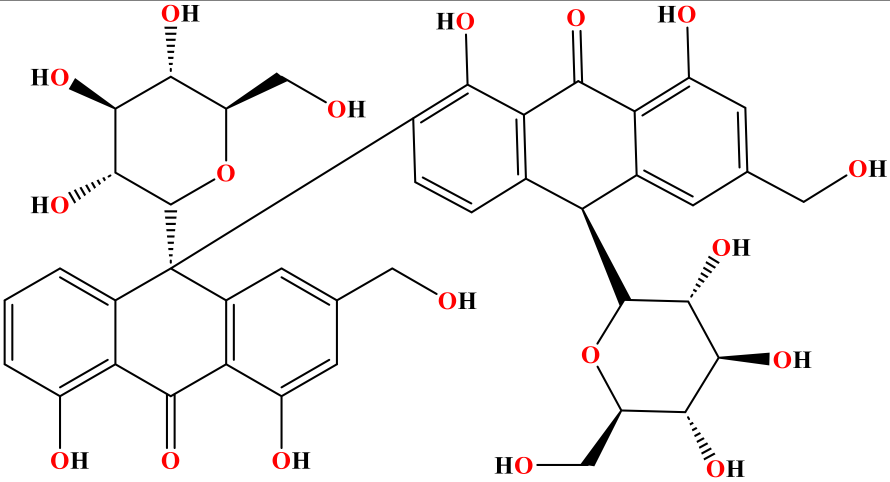

M48	Aloindimer B	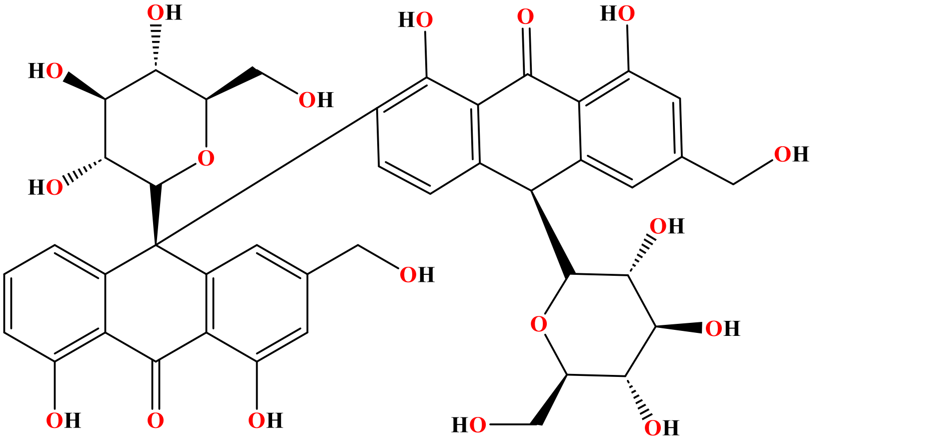
M49	Aloindimer C	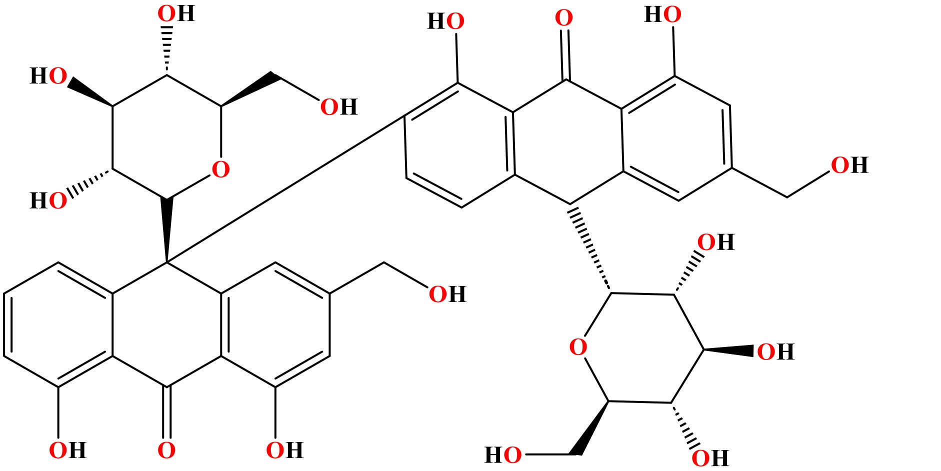
M50	Aloindimer D	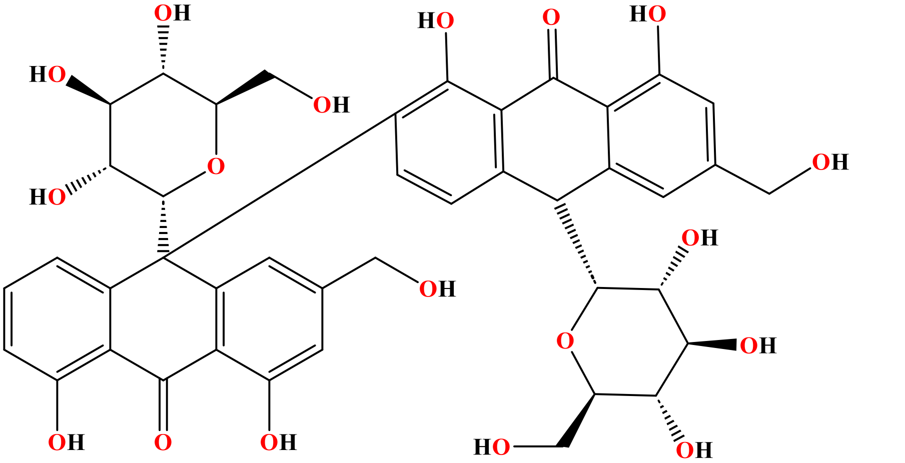
M51	Aloe-emodin-11-O-rhamnoside	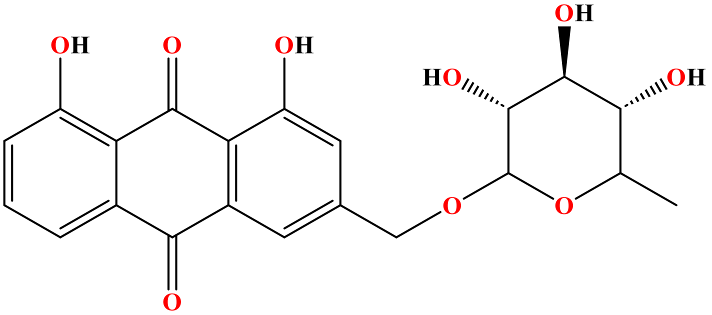

M52	Chrysophanol	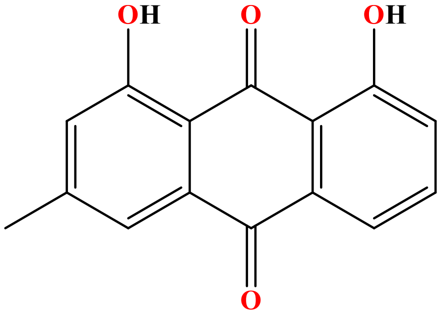
M53	Emodin	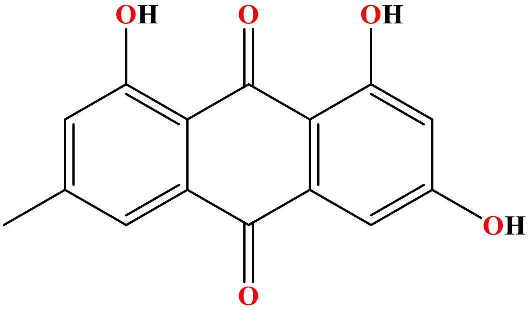
M54	Physcione	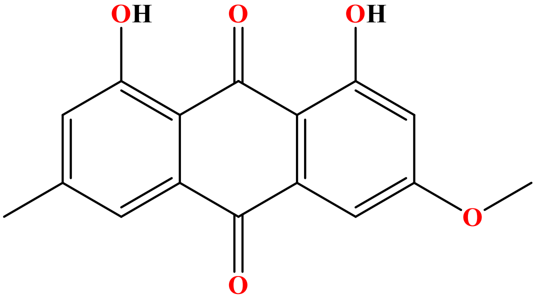
M55	Aloe-emodin	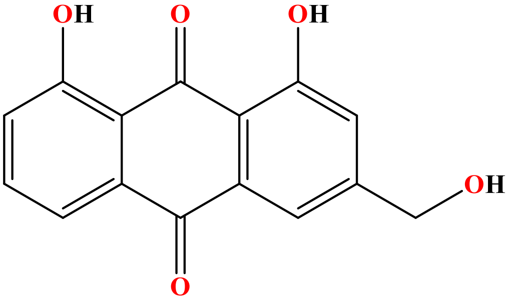

M56	Nataloe-emodin	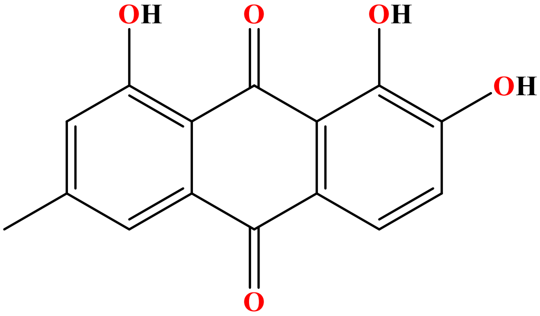

M57	Aloesaponarin I	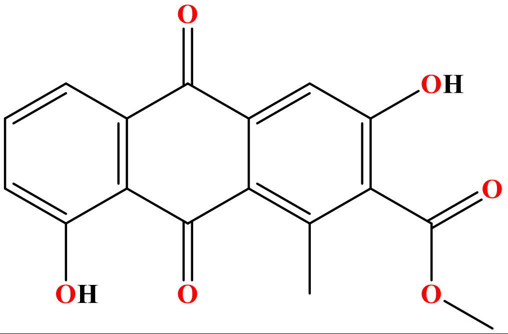
M58	Aloesaponarin II	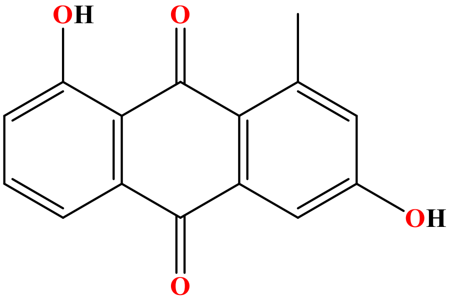
M59	Madagascine	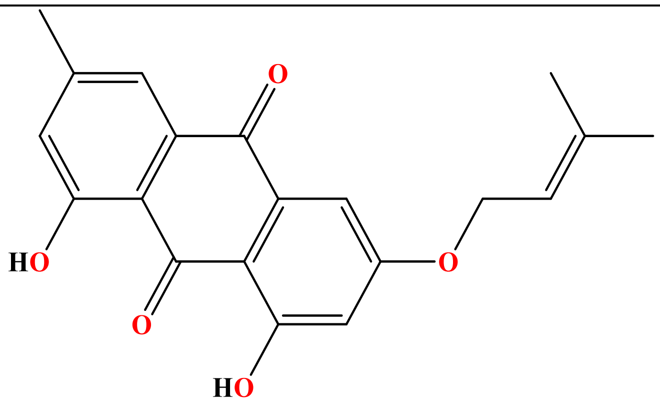
M60	3-Geranyloxyemodin	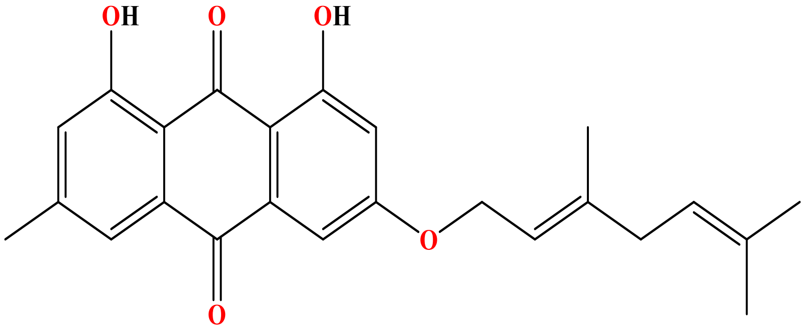
M61	Rhein	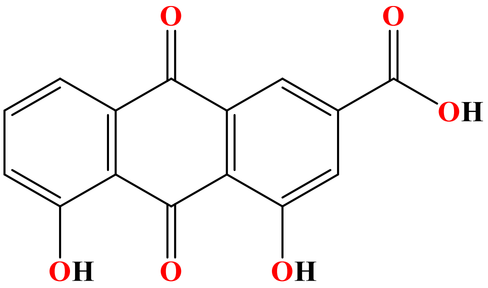

M62	Apigenin	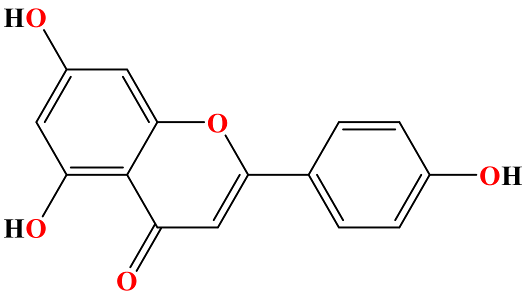
M63	Luteolin	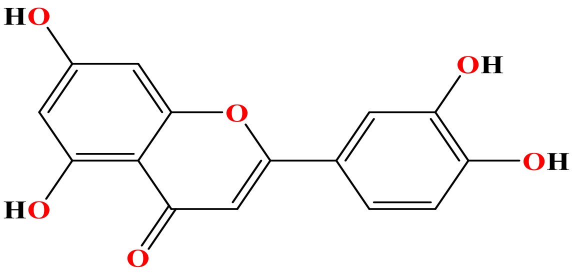
M64	Isovitexin	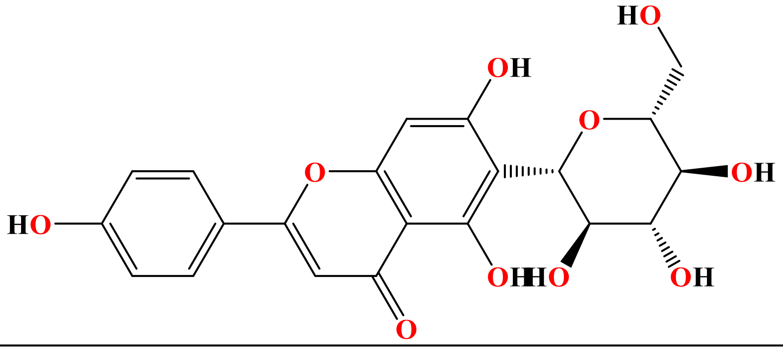
M65	Isoorientin	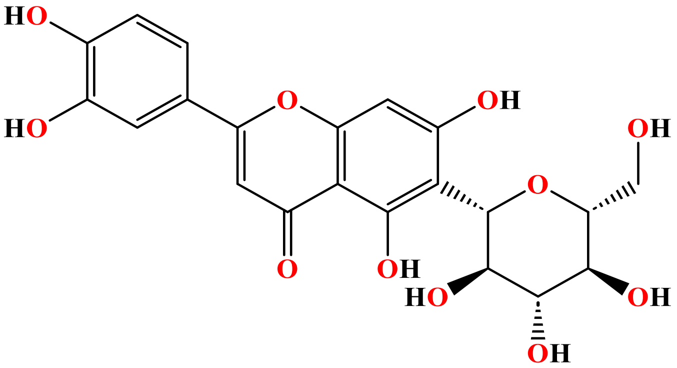
M66	Saponarin	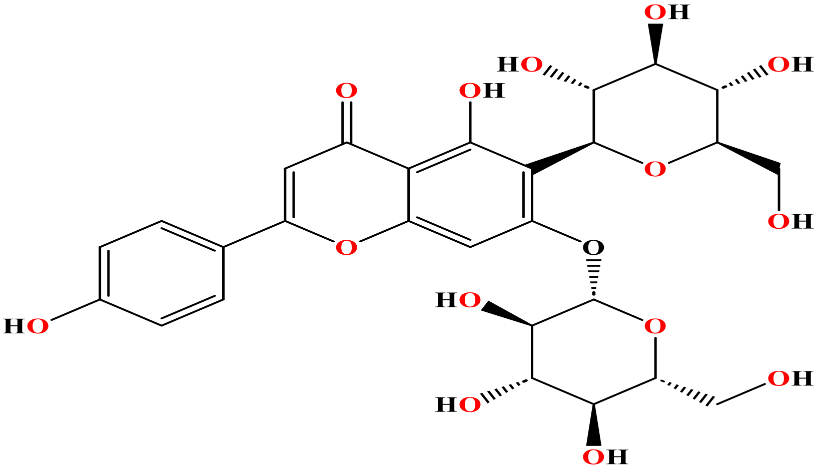

M67	Lutonarin	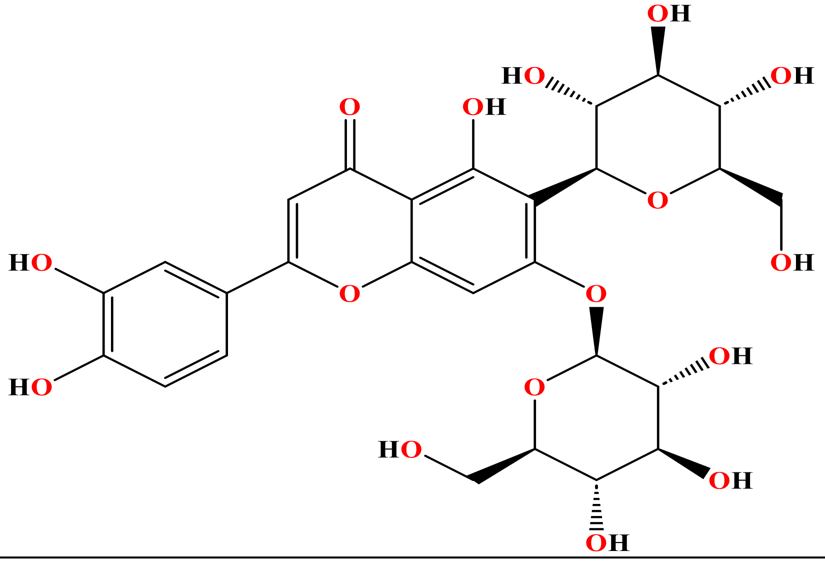
M68	Quercetin	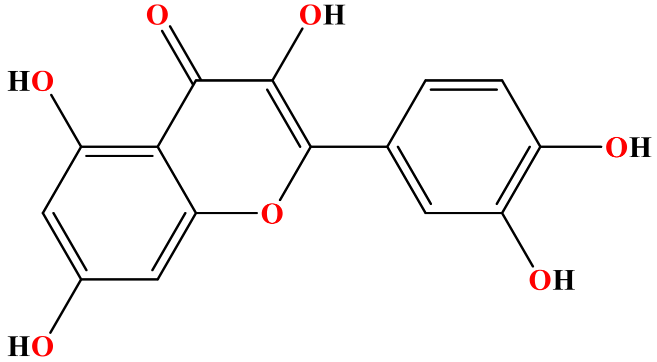
M69	Kaempferol	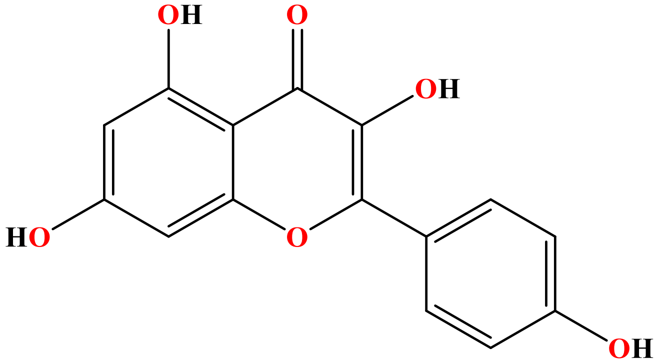
M70	Myricetin	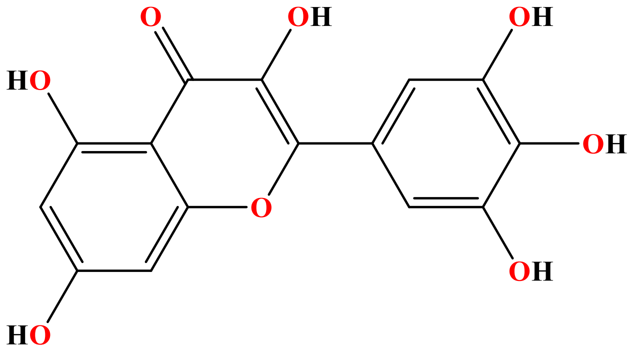

M71	Quercitrin	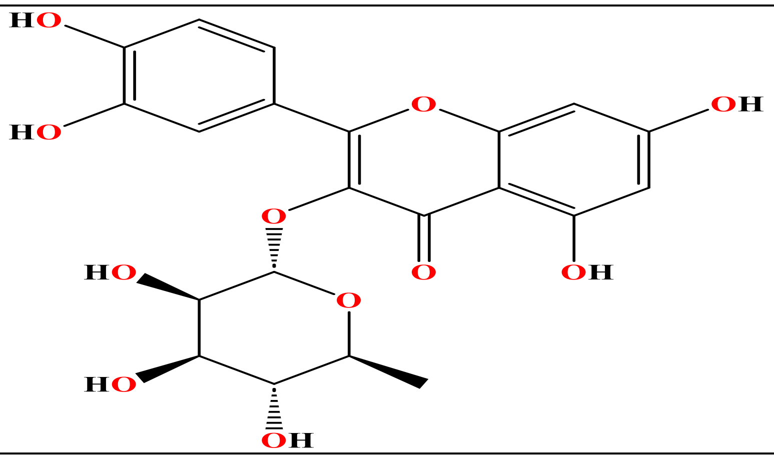
M72	Rutin	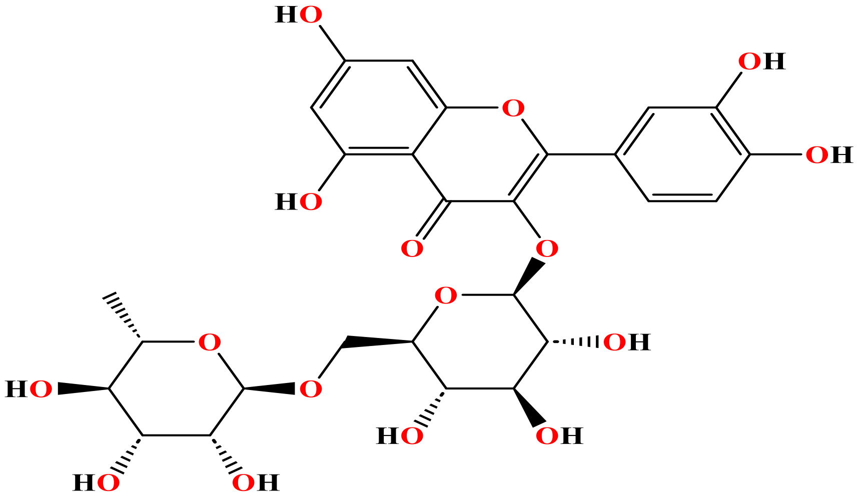
M73	Catechin	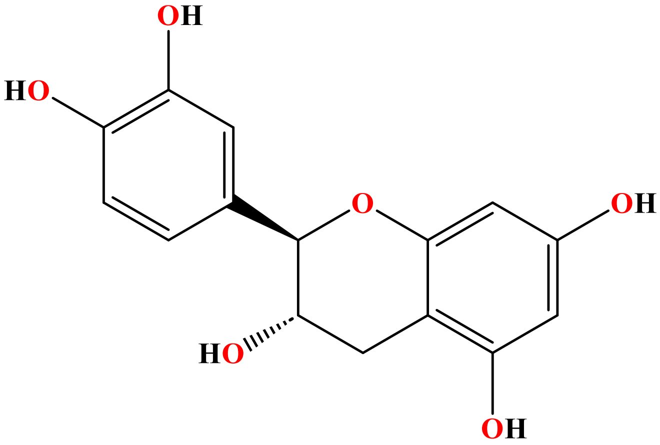
M74	Epicatechin	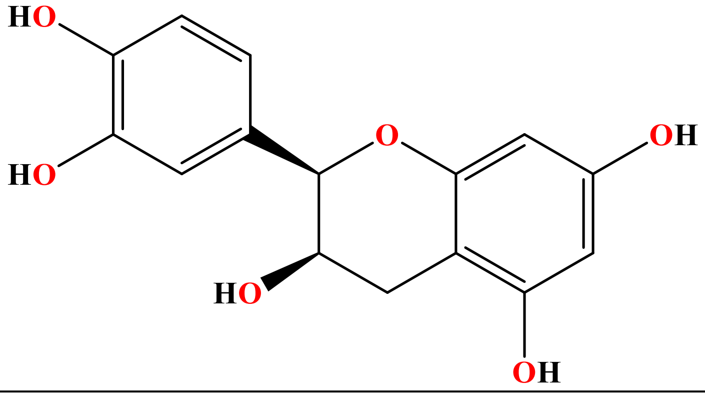

M75	Cinnamic acid	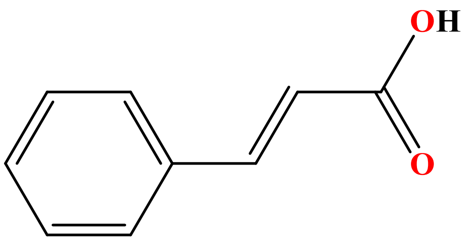
M76	P-coumaric	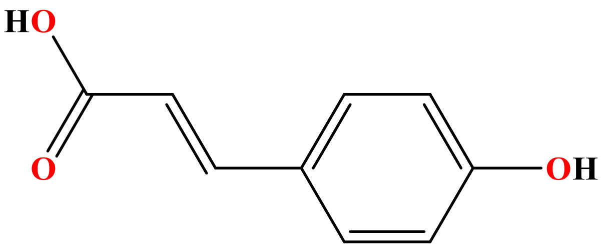
M77	Caffeic acid	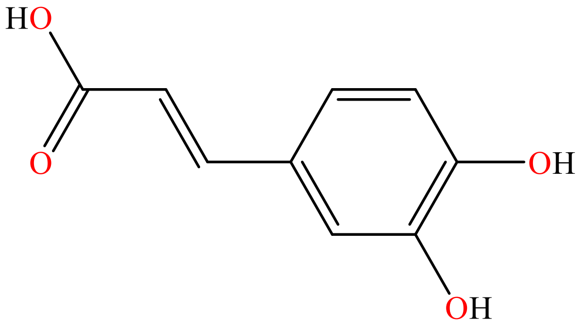
M78	Ferulic acid	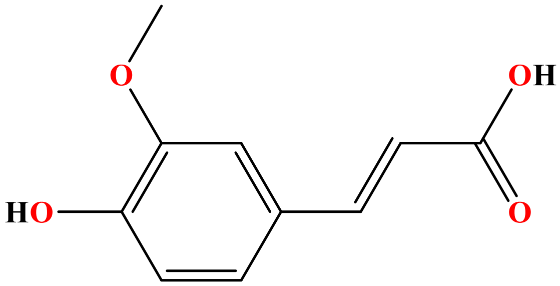
M79	Sinapic acid	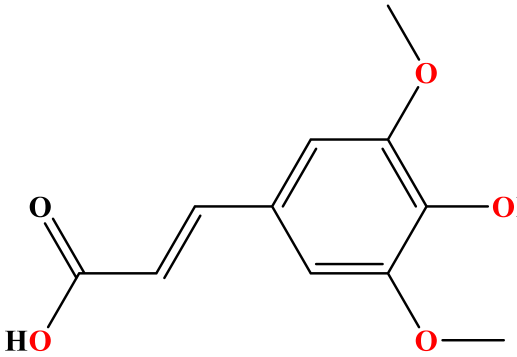

M80	5-p-coumaroylquinic	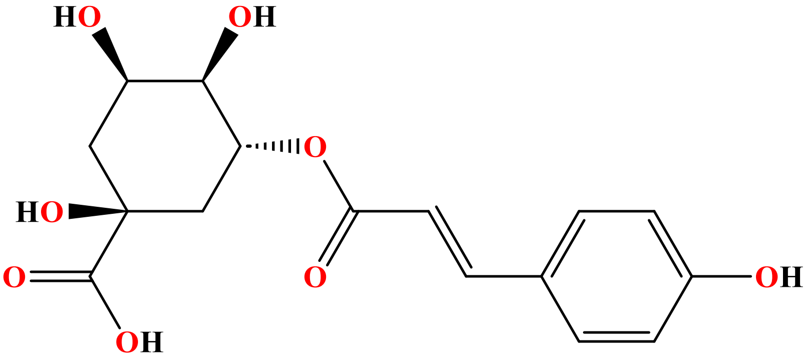
M81	Chlorogenic	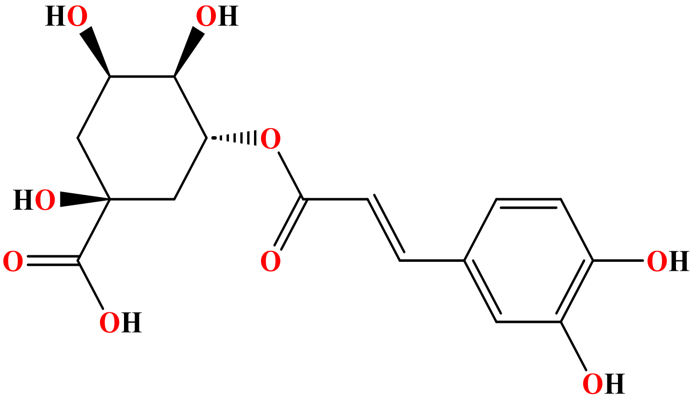
M82	5-feruloylquinic	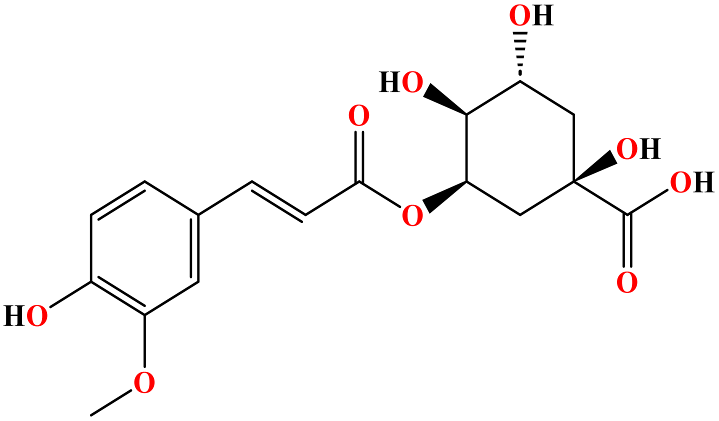
M83	Caffeoylshikimic	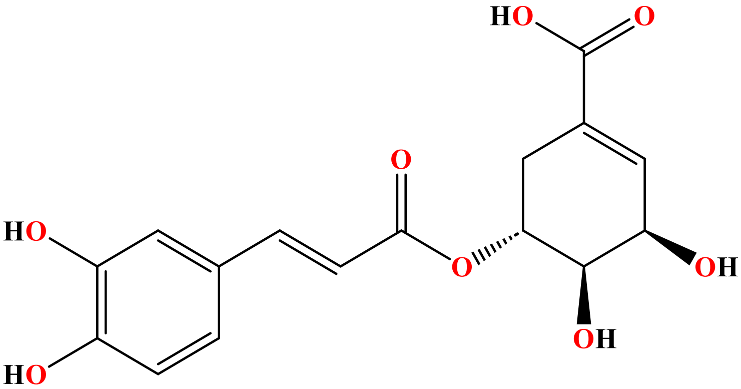

M84	5-p-cis-coumaroylquinic	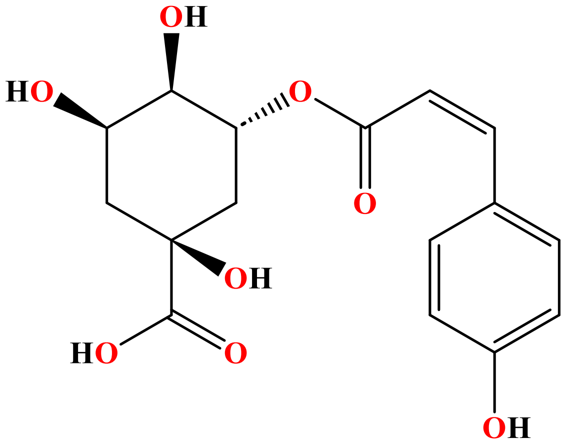
M85	3-(4-hydroxyphenyl) propanoic acid	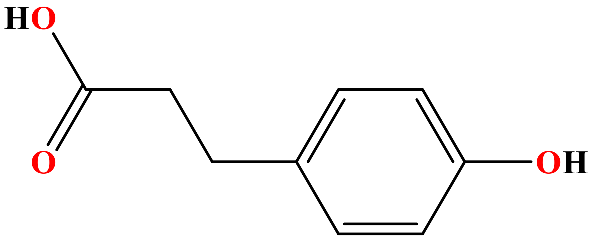
M86	Methyl 3-(4-hydroxyphenyl) propionate	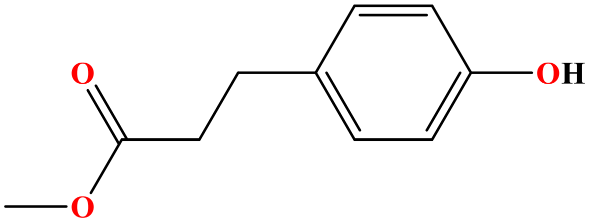
M87	7-demethylsiderin	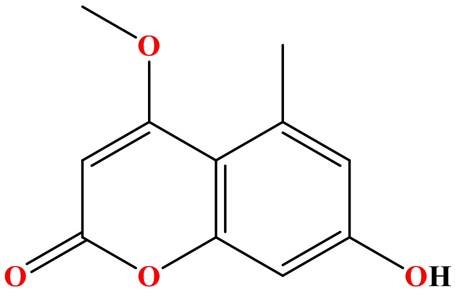
M88	Feralolide	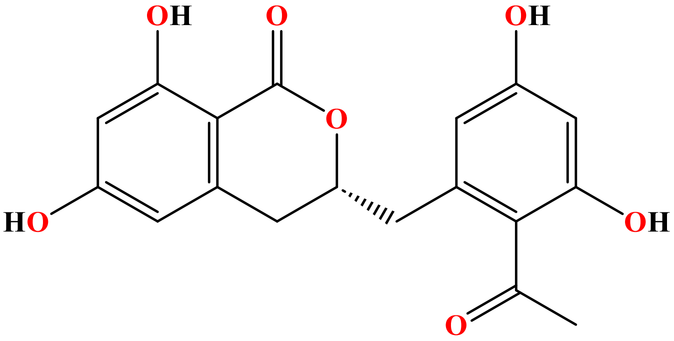

M89	Dihydrocoumarin	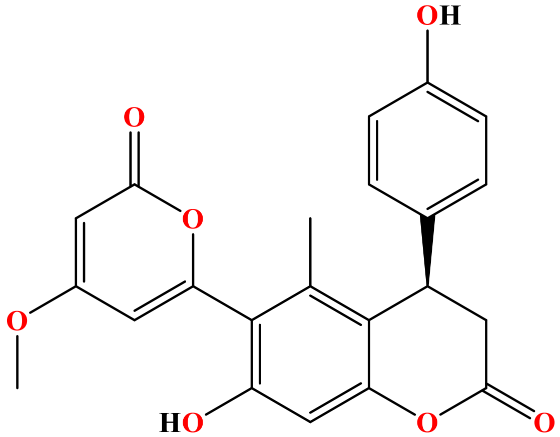
M90	Dihydrocoumarin ethyl ester	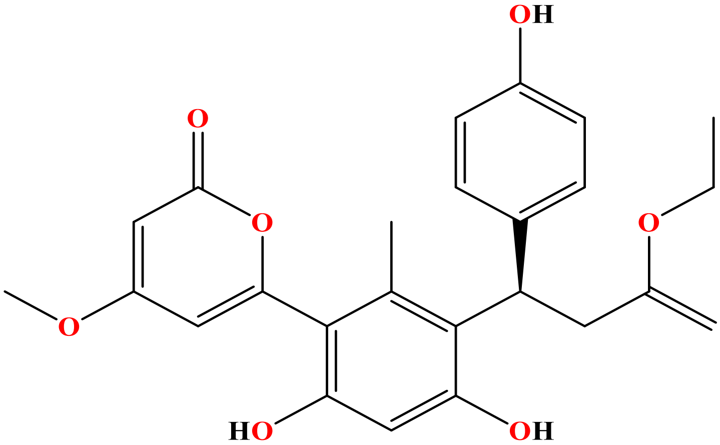
M91	Aloenin A	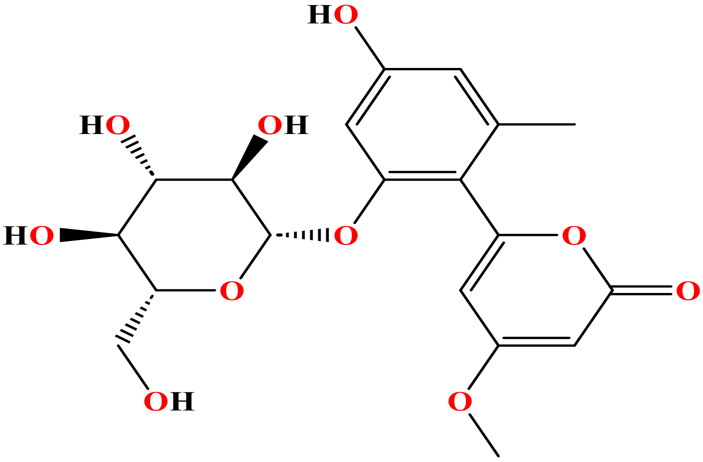

M92	Aloenin B	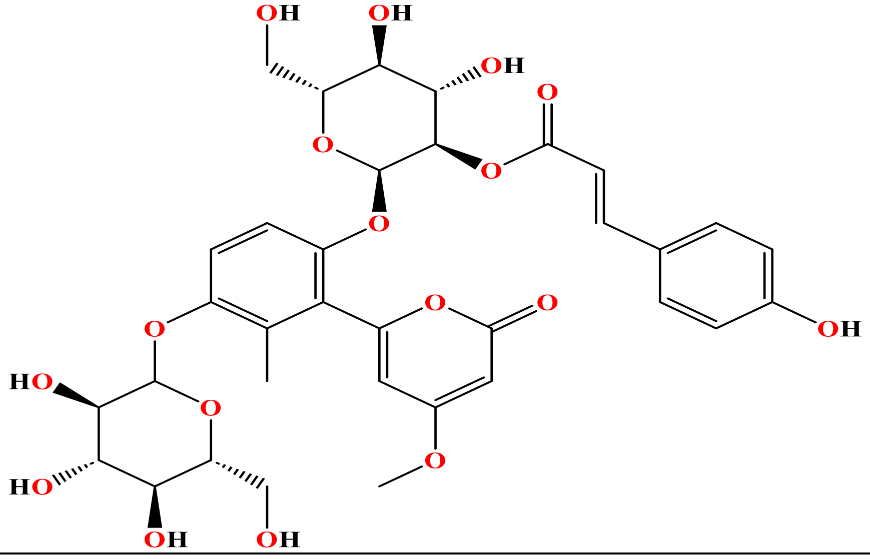
M93	P-coumaroyl aloenin	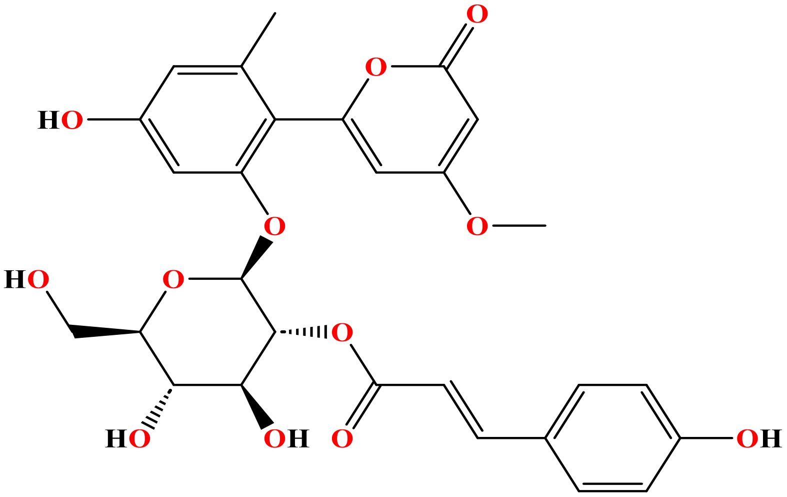
M94	Aloveroside A	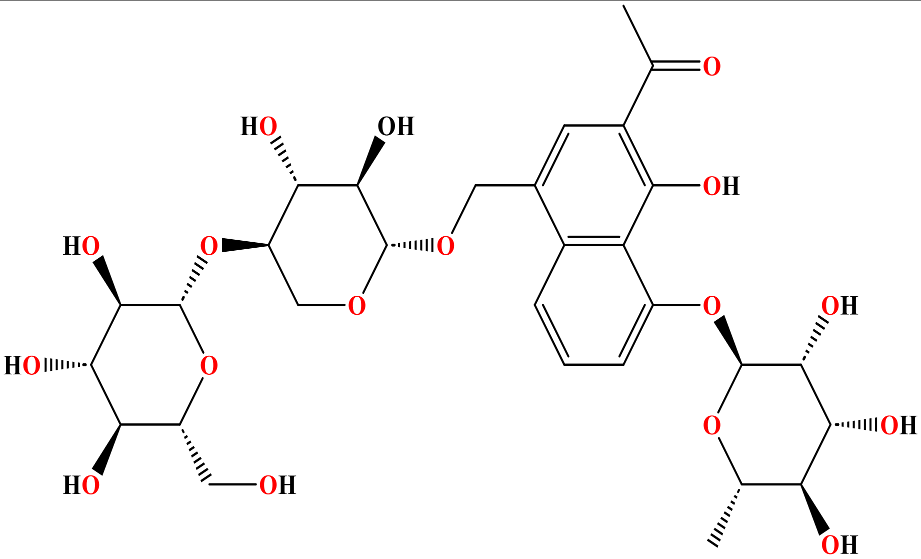

M95	Feroxidin	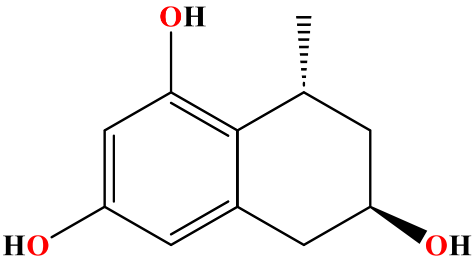
M96	1-(2,4-dihydroxy-6-methylphenyl) ethanone	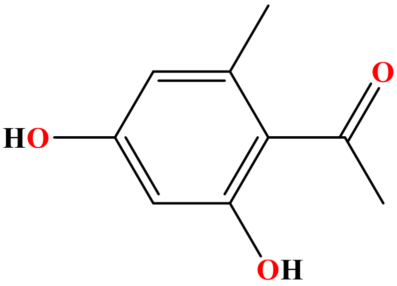
M97	P-anisaldehyde	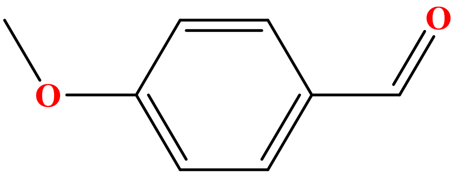
M98	Salicylaldehyde	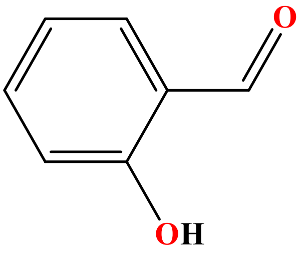
M99	P-cresol	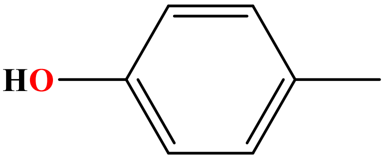

M100	Pyrocatechol	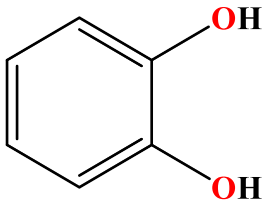
M101	Gentisic acid	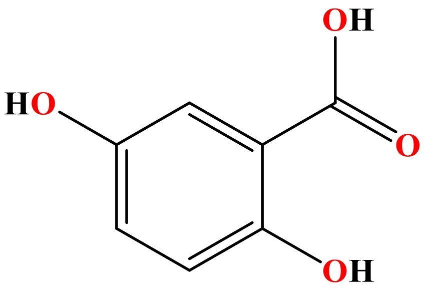
M102	Gallic acid	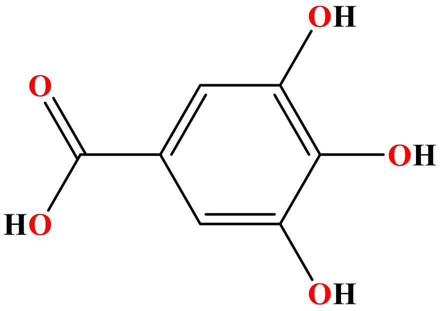
M103	Vanillic acid	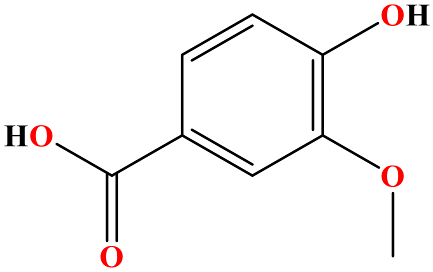
M104	Syringic acid	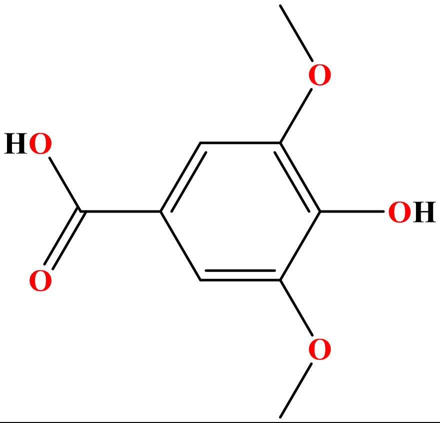

M105	Ascorbic acid	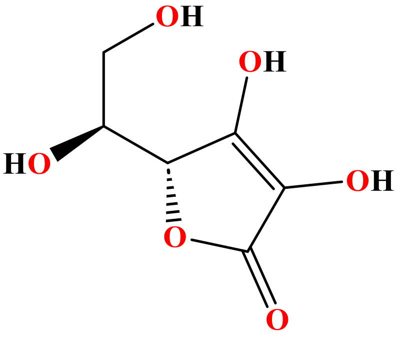
M106	Cycloartanol	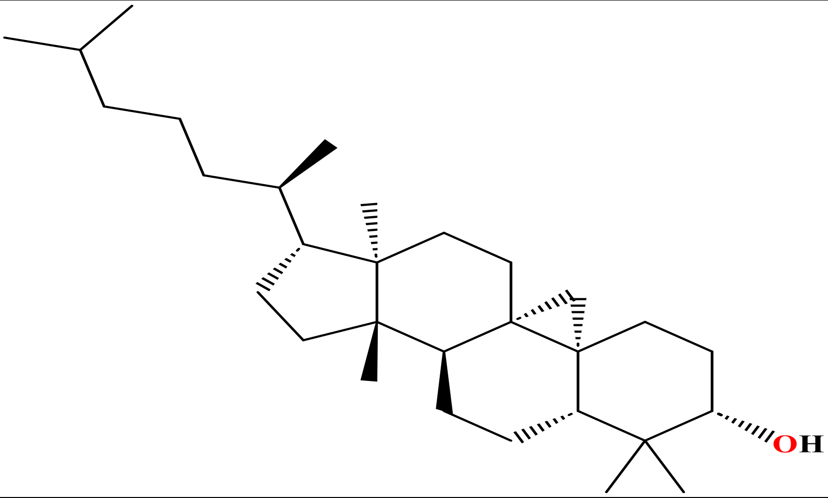
M107	24-methylene-cycloartanol	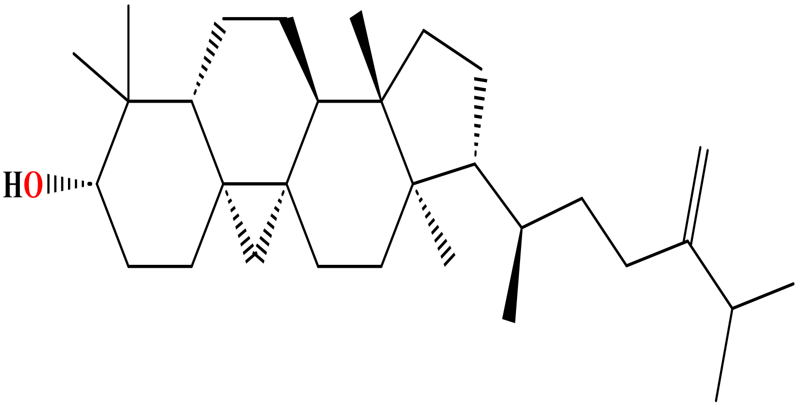
M108	Lophenol	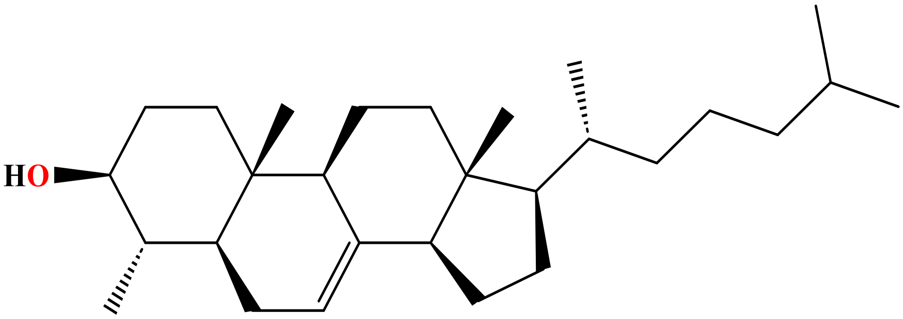

M109	24-methyl-lophenol	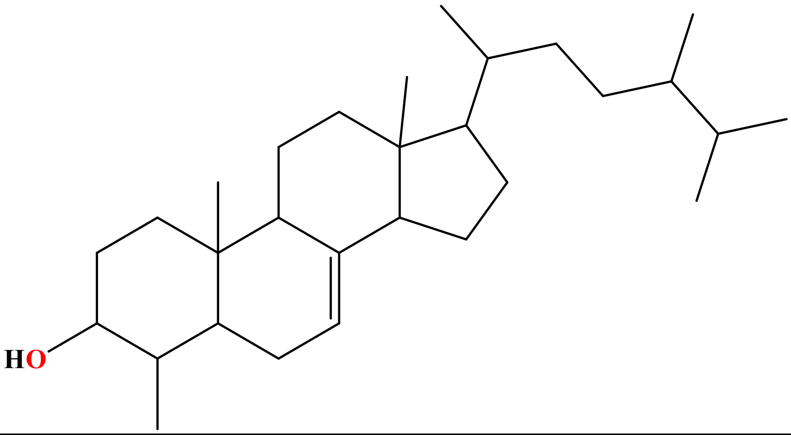
M110	24-ethyl-lophenol	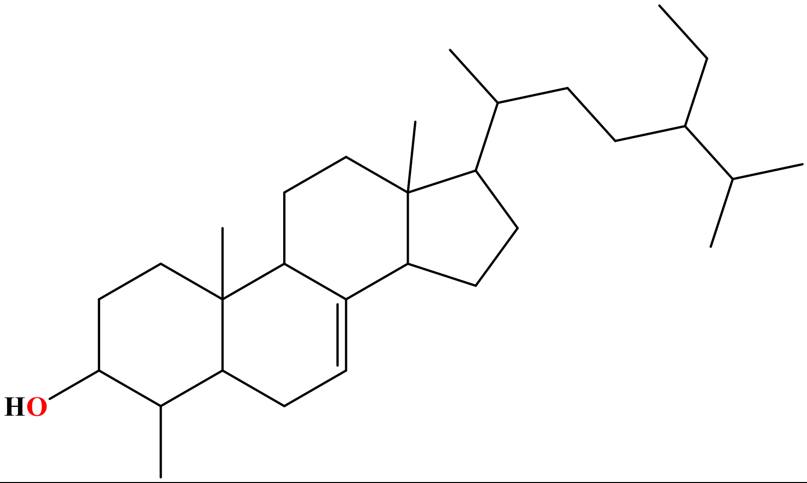

Table S2: Molecular docking scores of aloe vera-derived compounds against target proteins in atopic dermatitis

ID	5EH1	5mj3	4RG2	2BDF	3O96	1NME
M1	-6,6	-6.1	-7.3	-7.8	-8.4	-6.3
M2	-6,5	-6.4	-6.5	-8.3	-10.3	-7.3
M3	-7,2	-6.3	-7.4	-8.4	-8.9	-6.6
M4	-6,4	-5.9	-6.5	-8.0	-9.1	-6.6
M5	-7	-6.2	-7.4	-8.1	-8.5	-6.5
M6	-6,2	-6.2	-6.6	-7.9	-9.3	-6.7
M7	-7,1	-6.2	-6.7	-8.4	-9.2	-7.1
M8	-7,3	-7.4	-7.3	-9.1	-9.0	-7.9
M9	-8,3	-7.7	-7.7	-9.0	-8.8	-8.1
M10	-6,9	-6.0	-6.4	-8.5	-10.5	-6.5
M11	-7,1	-7.1	-7.0	-7.7	-8.8	-7.6
M12	-7,1	-6.6	-7.1	-8.1	-8.3	-8.0
M13	-7,1	-6.5	-7.4	-7.9	-8.7	-8.4
M14	-7,1	-6.9	-7.3	-7.9	-8.4	-7.1
M15	-7,1	-6.6	-7.5	-8.3	-8.4	-7.7
M16	-7,2	-6.5	-7.4	-8.0	-9.6	-7.6
M17	-7,1	-6.3	-6.8	-8.4	-7.5	-6.9
M18	-6,7	-6.8	-6.8	-7.3	-7.7	-7.3
M19	-6,5	-6.3	-7.0	-8.4	-8.2	-7.1
M20	-6,8	-5.8	-7.1	-7.7	-9.8	-6.3
M21	-7	-7.8	-7.9	-8.9	-8.6	-8.4
M22	-7,2	-7.4	-7.5	-8.5	-8.5	-7.9
M23	-6,9	-7.1	-7.5	-7.7	-7.9	-7.6
M24	-6,8	-6.6	-6.9	-8.0	-7.9	-7.3
M25	-7,5	-7.2	-7.5	-7.2	-8.8	-7.3
M26	-7,2	-7.1	-7.3	-8.7	-9.0	-7.5
M27	-7,3	-6.8	-6.9	-9.1	-7.8	-7.8
M28	-7,6	-6.4	-6.6	-7.6	-9.0	-8.0
M29	-7,1	-6.2	-6.6	-7.4	-7.8	-7.7
M30	-7,2	-6.9	-7.2	-8.0	-9.0	-7.4
M31	-7,2	-6.5	-7.0	-7.1	-8.2	-7.0
M32	-7,2	-6.5	-6.9	-7.3	-8.1	-7.1
M33	-7	-7.1	-6.9	-8.2	-8.4	-7.1
M34	-6,9	-6.0	-7.2	-8.6	-8.1	-7.5
M35	-7,8	-7.0	-6.8	-8.0	-7.9	-7.0
M36	-8,2	-7.0	-7.7	-8.2	-9.5	-8.3
M37	-7,9	-7.3	-8.1	-8.6	-7.9	-9.3
M38	-7,3	-6.6	-7.4	-8.2	-8.1	-7.5
M39	-7,6	-6.6	-6.9	-7.5	-8.2	-7.0
M40	-6,8	-6.3	-7.3	-8.1	-7.6	-7.3
M41	-7,5	-6.5	-6.7	-7.2	-7.4	-7.0
M42	-7,5	-7.1	-7.3	-8.4	-8.2	-7.6
M43	-7	-6.6	-7.0	-9.1	-7.7	-7.6
M44	-7,3	-7.2	-7.8	-8.7	-8.9	-7.9
M45	-8,4	-8.6	-9.3	-9.9	-9.7	-8.5

**M46**	-8,4	-7.9	-9.5	-10.2	-9.2	-8.6
**M47**	-9,1	-7.8	-8.0	-9.8	-9.3	-8.9
**M48**	-9,4	-7.7	-8.9	-9.5	-9.4	-9.4
**M49**	-9,4	-7.7	-8.9	-9.5	-9.4	-9.4
**M50**	-9,4	-7.7	-8.9	-9.5	-9.4	-9.4
**M51**	-7,8	-6.8	-7.8	-9.7	-11.5	-8.1
**M52**	-7,1	-6.5	-7.5	-9.0	-10.3	-6.9
**M53**	-7	-6.7	-7.6	-9.0	-10.4	-6.7
**M54**	-7	-6.8	-7.2	-9.0	-10.2	-6.6
**M55**	-6,5	-6.4	-7.3	-8.8	-10.3	-6.7
**M56**	-7	-6.7	-7.1	-8.9	-10.2	-6.9
**M57**	-6,4	-7.1	-7.3	-9.0	-10.3	-6.7
**M58**	-6,4	-6.5	-7.9	-9.1	-10.3	-6.9
**M59**	-7,3	-7.4	-7.5	-9.8	-10.5	-7.4
**M60**	-6,8	-6.8	-8.1	-9.8	-10.5	-7.8
**M61**	-6,6	-6.8	-7.6	-9.1	-10.4	-7.0
**M62**	-6,9	-6.5	-7.0	-8.3	-9.5	-7.1
**M63**	-6,9	-6.4	-6.8	-8.5	-9.8	-7.4
**M64**	-7,8	-7.7	-7.6	-8.8	-10.6	-8.1
**M65**	-8,3	-6.6	-7.4	-8.6	-11.0	-8.1
**M66**	-7,6	-7.1	-7.4	-9.3	-9.1	-8.6
**M67**	-8,2	-8.2	-8.3	-9.5	-9.9	-8.8
**M68**	**-8.1**	**-7.9**	**-8.2**	**-8.5**	**-8.2**	**-8**
**M69**	**-8**	**-7.6**	**-8.3**	**-8.1**	**-8.4**	**-7.9**
**M70**	-7,1	-6.0	-6.5	-8.6	-9.6	-7.6
**M71**	-7,4	-7.0	-7.1	-8.9	-10.7	-7.0
**M72**	-7,7	-6.9	-7.2	-8.0	-9.5	-8.0
**M73**	-7	-6.0	-7.3	-8.3	-9.6	-7.7
**M74**	-6,6	-6.5	-7.1	-8.2	-9.0	-7.2
**M75**	-5	-4.7	-6.3	-5.9	-6.5	-5.4
**M76**	-5,6	-4.7	-5.8	-6.0	-6.8	-6.1
**M77**	-5,7	-5.0	-5.6	-6.3	-6.9	-6.1
**M78**	-5,8	-4.7	-5.8	-6.2	-6.9	-6.1
**M79**	-5,6	-4.9	-5.4	-6.1	-6.7	-5.4
**M80**	-6,8	-6.5	-7.0	-7.5	-10.4	-6.9
**M81**	-6,8	-6.0	-6.9	-7.6	-9.9	-7.1
**M82**	-6,9	-6.2	-7.0	-8.8	-8.5	-7.0
**M83**	-7,1	-6.7	-6.8	-8.2	-9.2	-7.1
**M84**	-6,3	-6.4	-6.0	-7.5	-9.7	-6.3
**M85**	-5,4	-5.0	-6.0	-6.1	-6.6	-5.9
**M86**	-4,9	-5.0	-5.8	-6.0	-6.4	-5.2
**M87**	-5,5	-5.3	-6.5	-7.1	-7.8	-5.7
**M88**	-7,2	-7.0	-7.2	-8.1	-10.2	-6.9
**M89**	-7,1	-7.1	-7.6	-8.4	-10.0	-7.1
**M90**	-6,5	-6.0	-6.3	-7.5	-6.9	-7.0
**M91**	-7,1	-6.2	-7.3	-8.0	-9.5	-7.2
**M92**	-7,2	-7.0	-7.4	-8.3	-8.8	-9.0
**M93**	-7,5	-7.5	-7.7	-8.1	-8.0	-7.8

**M94**	-8,4	-9.1	-7.3	-9.2	-9.3	-8.5
**M95**	-5,5	-5.1	-6.8	-7.1	-7.5	-5.7
**M96**	-4,9	-4.6	-5.6	-5.9	-6.4	-5.0
**M97**	-4,4	-4.4	-5.8	-5.1	-5.7	-4.7
**M98**	-4,6	-4.4	-5.2	-5.2	-5.4	-4.6
**M99**	-4,5	-4.1	-5.1	-4.9	-5.6	-4.7
**M100**	-4,4	-4.2	-4.8	-4.7	-5.5	-4.3
**M101**	-4,8	-4.7	-5.7	-5.6	-6.0	-5.0
**M102**	-4,7	-4.9	-5.8	-5.6	-6.0	-5.0
**M103**	-4,9	-4.6	-5.8	-5.7	-6.0	-5.3
**M104**	-4,8	-4.5	-5.6	-5.7	-6.2	-4.9
**M105**	-4,8	-4.6	-4.9	-5.4	-5.3	-4.8
**M106**	-6,8	-6.8	-7.1	-7.8	-10.9	-6.9
**M107**	-7,4	-7.2	-7.8	-7.6	-11.5	-8.0
**M108**	-6,5	-7.2	-7.9	-8.2	-10.6	-7.3
**M109**	-6,8	-7.1	-6.7	-8.0	-10.4	-7.9
**M110**	-6,1	-7.1	-6.8	-7.3	-8.6	-7.8
**Natif ligand**	-5,7	-2,9	-7,3	-9,4	-9,6	-5,1
**Reference**	-8	-7,2	-6,1	-6,8	-9,4	-6,7
